# Insect and mite pests of pepino (*Solanum muricatum* Ait.) in Japan

**DOI:** 10.3897/BDJ.7.e36453

**Published:** 2019-08-13

**Authors:** Tadashi Ishikawa, Ken Takahata

**Affiliations:** 1 Laboratory of Entomology, Faculty of Agriculture, Tokyo University of Agriculture, Atsugi-shi, Kanagawa, Japan Laboratory of Entomology, Faculty of Agriculture, Tokyo University of Agriculture Atsugi-shi, Kanagawa Japan; 2 Laboratory of Vegetables, Faculty of Agriculture, Tokyo University of Agriculture, Atsugi-shi, Kanagawa, Japan Laboratory of Vegetables, Faculty of Agriculture, Tokyo University of Agriculture Atsugi-shi, Kanagawa Japan

**Keywords:** sweet cucumber, pest management, *Tetranychus urticae*, *Thrips tabaci*, *Bemisia tabaci*

## Abstract

To further increase the basic knowledge regarding the establishment of pest control for pepino (*Solanum muricatum* Ait.), we conducted surveys of pepino pests in Japan. Thirty-four insect and four mite species were recognized as pests of pepino plants in the present study. Including the results of previous studies, a total of 41 species of insects and mites have been reported as pests of pepino plants in Japan. Three species, namely onion thrips (*Thrips tabaci*), two-spotted spider mites (*Tetranychus urticae*), and cotton whiteflies (*Bemisia tabaci*), are likely the most important insect and mite pests of pepino plants, because they were collected from more than half of the study sites and were much more abundant on pepino plants than the other pest species.

## Introduction

Pepino (*Solanum muricatum* Ait., the Spanish name for sweet cucumber) is a solanaceous plant cultivated as a fruit crop and native to the Andes. To date, 22 insect and three mite species have been recorded as pests of pepino worldwide (excluding Japan). Seven of them, inclusive of the two-spotted spider mite *Tetranychus urticae* Koch, 1836, are regarded to be the most important among the pests of pepino (Larraín 2002; Galbreath and Clearwater 1983; Akyazi 2012). In 2016, our research team began a research project aimed at producing high quality and flavorsome pepino fruits, whose soluble solids content was rather low in the Japanese fruits (Sakata 2011). In order to establish solid pest control in its commercial cultivation and to produce high quality and stable pepino fruits, our research team has tried to comprehensively elucidate the pests of pepino in the project.

To date, 13 insect and mite species have been recorded in Japan as pests of pepino (Kim et al. 2017). However, few studies have been conducted on pests of pepino plants in Japan. The reason for this may be that the number of pests of pepino plants recognized in Japan is rather low compared to those of other popular solanaceous crops such as tomato (*S. lycopersicum*), eggplant (*S. melongena*), potato (*S. tuberosum*), and green pepper (*Capsicum annuum*) (The Japanese Society of Applied Entomology and Zoology 2006). This low number of pests is attributable to the small area in which studies have been conducted on pepino, which has a radius of 250 m at most (Kim et al. 2017). In order to develop an accurate understanding of pests of pepino plants, it is necessary to conduct research across an extensive area of Japan.

In order to expand the basic knowledge required for the establishment of pest control for pepino plants, we conducted investigations of pepino pests in Japan in the experimental fields of our university, Tokyo University of Agriculture, as well as on farms and in garden centers in Japan. This study was conducted under a project for regional development titled ‘Launching of Nodai-branded Pepino Crop’ conducted by the Faculty of Agriculture, Tokyo University of Agriculture (Kim et al. 2017). This paper documents the results of our field surveys of pests of pepino plants in Japan after the latest report by Kim et al. (2017), with a brief discussion on pests of importance to the cultivation of pepino in Japan.

## Materials and methods

### Study sites

**Figure 1.**
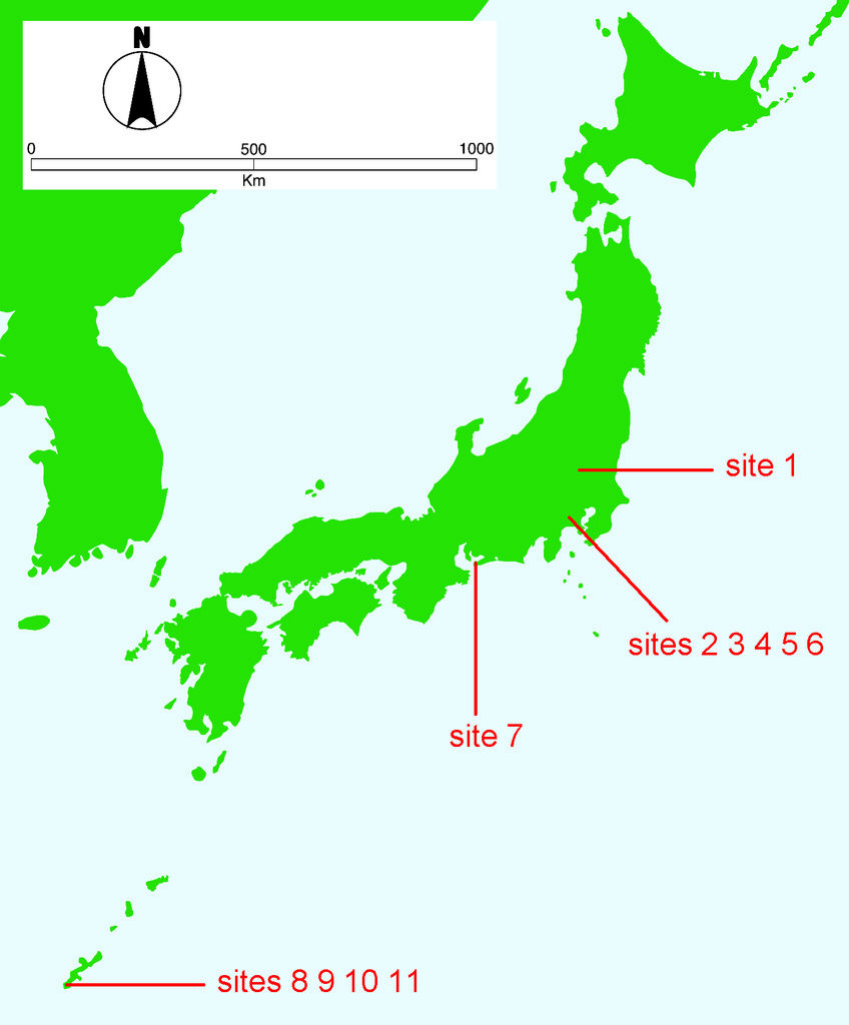
Locations of the 11 study sites in Japan.

This study was conducted at 11 sites in Japan (Fig. 1). Of these, sites 1–7 are in a warm-temperate climate zone, and sites 8–11, on Okinawa Island, are in a subtropical climate zone. The sites are as follows: Site 1 (Fig. 2a): a greenhouse located in Ookubo, Tochigi-shi, Tochigi Prefecture (36.439N 139.668E; 93 meters above sea level (m a.s.l.)), surrounded by hills and vegetable fields. Approximately 10 potted pepino plants were cultivated at site 1. Site 2 (Fig. 2b): an open field located in Nurumizu, Atsugi-shi, Kanagawa Prefecture (35.433N 139.348E; 43 m a.s.l.), surrounded by residential quarters and a woody and grassy park. Approximately 40 pepino plants were cultivated at site 2. Site 3 (Fig. 2c): an open field (with a roof against rain) located in Hase, Atsugi-shi, Kanagawa Prefecture (35.432N 139.346E; 49 m a.s.l.), surrounded by residential quarters and a woody and grassy park. Approximately 20 pepino plants were cultivated at site 3. Site 4 (Fig. 2d): a greenhouse located in northern Funako, Atsugi-shi, Kanagawa Prefecture (35.431N 139.350E; 27 m a.s.l.), surrounded by residential quarters and a woody and grassy park. Approximately 60 potted pepino plants were cultivated at site 4. Site 5 (Fig. 2e): a greenhouse located in southern Funako, Atsugi-shi, Kanagawa Prefecture (35.429N 139.349E; 42 m a.s.l.), surrounded by residential quarters and a woody and grassy park. Approximately 400 potted pepino plants were cultivated at site 5. Site 6 (Fig. 2f): a greenhouse located in San-nomiya, Isehara-shi, Kanagawa Prefecture (35.400N 139.282E; 62 m a.s.l.), surrounded by vegetable fields. Approximately 100 potted pepino plants were cultivated at site 6. Site 7 (Fig. 3a): a greenhouse located in Koshiozu, Tahara-shi, Aichi Prefecture (34.600N 137.097E; 27 m a.s.l.), surrounded by vegetable fields and hills. Approximately 1000 potted pepino plants were cultivated at site 7. Site 8 (Fig. 3b): an open field located in Miyahira, Haebaru-cho, Okinawa Prefecture (26.189N 127.735E; 34 m a.s.l.), surrounded by vegetable fields. Approximately 20 pepino plants were cultivated at site 8. Site 9 (Fig. 3c): an open field located in Kyan, Haebaru-cho, Okinawa Prefecture (26.186N 127.736E; 18 m a.s.l.), surrounded by vegetable fields. Approximately 20 pepino plants were cultivated at site 9. Site 10 (Fig. 3d): a garden center located in Inamine, Nanjo-shi, Okinawa Prefecture (26.172N 127.734E; 43 m a.s.l.), surrounded by residential quarters. Approximately 25 potted pepino plants were displayed for sale at site 10. Site 11 (Fig. 3e): a garden center located in Takahira, Nanjo-shi, Okinawa Prefecture (26.171N 127.737E; 34 m a.s.l.), surrounded by residential quarters. Approximately 15 potted pepino plants were displayed for sale at site 11.

### Sampling methods

All specimens were collected by looking at or beating the leaves, branches and fruits of pepino plants. A total of more than 80 collections were performed in the 11 study sites (once at sites 1, 6, 7, 10, and 11; three times at site 4; four times at sites 8 and 9; nine times at site 5; 24 times at site 3; and more than 30 times at site 2) from February 24th, 2017 to March 14th, 2019. Our sampling period followed that of Kim et al. (2017), with two exceptions, as unidentified specimens collected on October 26th and November 23rd, 2016 represented the species not found in this main survey. Each of the collections was conducted for a maximum of three hours during the daytime by one or two persons. The collected insects and mites were killed immediately after capture, using ethyl acetate. Aphids, lepidopteran larvae, and mites were fixed in plastic bottles filled with 70–80% ethanol. All specimens, which were killed with ethyl acetate and fixed with ethanol, were prepared as dry mounted, slide-mounded, or ethanol preserved for morphological examination. Slide-mounted specimens were prepared with the following procedure: specimens were macerated in a hot 5–7% KOH solution for 5 minutes; macerated specimens were washed in distilled water for a few minutes; washed specimens were moved from distilled water onto a drop of Neo-Sigaral (balsam-like liquid for easy preparation method; Shiga-Konchu-Fukyusha, Tokyo, Japan) on the middle of a glass slide, and then covered gently with a 12 mm (15 mm for larger specimen) cover glass.

### Identification methods

Identification of insect and mite specimens was performed using stereoscopic microscopes (Olympus SZ60 and Olympus SZX16, Tokyo, Japan) and optical microscopes (Olympus BH-2 and Olympus BX41, Tokyo, Japan) by Tadashi Ishikawa, Yoshihiro Yamada, and Naoki Kaneko according to the following studies: Kawai (1980), Dworakowska (1982), Moritsu (1983), Kimoto and Takizawa (1994), Iwasaki et al. (2000), Yasunaga et al. (2001), Umeya and Okada (2003), Furukawa (2005), Orthopterological Society of Japan (2006), Matsumoto (2008), Ehara and Gotoh (2009), Yasuda et al. (2010), Japan Plant Protection Association (Ed) (2011), Kobayashi and Matsumoto (2011), Harada and Takizawa (2012), Ishikawa et al. (2012), Okajima and Araya (2012), Tanaka and Uesato (2012), Yasuda et al. (2012), Carapia Ruiz and Castillo-Gutiérrez (2013), Masumoto and Okajima (2013), Yasuda et al. (2014), Aoki (2015), Yasunaga et al. (2015), Sakamoto (2018), Tokumaru (2018), along with the original descriptions and/or redescriptions of corresponding species if necessary. Collected specimens were regarded as pests only in this paper if these were insects or mites that directly damaged pepino plants, were known as pests of pepino plants in the native range and introduced regions of pepino plants other than Japan (Galbreath and Clearwater 1983; Larraín 2002; Grinberg et al. 2005; Akyazi 2012), or were known as pests of major solanaceous crops such as tomato, eggplant, potato, and green pepper, in Japan, with reference to studies such as Umeya and Okada (2003) and The Japanese Society of Applied Entomology and Zoology (2006). All examined specimens are preserved in the Insect Collection (IC) at the Laboratory of Entomology, Tokyo University of Agriculture, Atsugi-shi, Kanagawa, Japan (LETUA).

## Results

In this study, 701 individual insects and mites belonging to 38 species were recognized as pests of pepino plants (Suppl. material 1). They consisted of 34 hexapod species belonging to 17 families in seven orders (which are classified into two classes, the Entognatha and the Insecta) and four mite species in one family and one order (Table 1). Of these 38 species, 35 have been known as pests of solanaceous crops such as tomato, eggplant, potato, and green pepper in Japan (Yasunaga et al. 1993; Yasunaga et al. 2001; Umeya and Okada 2003; Komine and Matsuo 2005; Yokohama Plant Protection Station 2005; Ono et al. 2006; The Japanese Society of Applied Entomology and Zoology 2006; Harada and Takizawa 2012). The remaining three species, the spotted grasshopper (*Atractomorpha sinensis* Bolivar, 1905), the black chafer (*Nigrotrichia kiotoensis* (Niijima et Kinoshita, 1923)), and the tussock caterpillar (*Orvasca taiwana* (Shiraki, 1913)), were newly recognized as pests of pepino plants.

## Discussion

Prior to the present study, the following 13 species of insects and mites were recognized as pests of pepino plants in Japan (Furusato 1984; Takahashi 1985; Takagi 1985; Kita 1986; Odagiri et al. 1986; Ozawa 1986; Kim et al. 2017, see also in Table 2): flower thrips (*Frankliniella intonsa* (Trybom, 1895)), cotton whiteflies (*Bemisia tabaci* (Gennadius, 1889)), greenhouse whiteflies (*Trialeurodes vaporariorum* (Westwood, 1856)), cotton aphids (*Aphis gossypii* Glover, 1877), solanum mealybugs (*Phenacoccus solani* Ferris, 1918), *Campylomma* plant bugs (*Campylomma livida* Reuter, 1885), tobacco flea beetles (*Epitrix hirtipennis* (Melsheimer, 1847)), vegetable leafminer (*Liriomyza sativae* Blanchard, 1938), potato tuberworms (*Phthorimaea operculella* (Zeller, 1873)), tobacco cutworms (*Spodoptera litura* (Fabricius, 1775)), cabbage loopers (*Trichoplusia ni* (Hübner, 1803)), broad mites (*Polyphagotarsonemus latus* (Banks, 1904)), and two-spotted spider mites (*Tetranychus urticae* Koch, 1836). In the present study, our surveys conducted in different locations in Japan revealed the presence of 38 species of insect and mite pests on pepino plants, as mentioned above (Table 1). Ten pest species were frequently recorded in the previous studies (Furusato 1984; Takagi 1985; Takahashi 1985; Kita 1986; Odagiri et al. 1986; Ozawa 1986; Kim et al. 2017) as well as in the present study. In addition, three species, namely solanum mealybugs, potato tuberworms, and broad mites, were not found in our surveys.

Including the results of the present study, a total of 41 species of insects and mites have been recorded as pests of pepino plants in Japan (Table 2). Therefore, 28 species are newly recorded as pepino pests in Japan. This increase in the number of pest species is likely the result of not only the longer sampling period in this study, but also the fact that more study sites were sampled in the present study than in the study by Kim et al. (2017), who undertook surveys for approximately one and a half years in three sites located within a radius of 250 m in Kanagawa Prefecture (sites 3, 4, and 5 in this study correspond to plots A, B, and C in Kim et al. (2017), respectively). In particular, the inclusion of study sites on Okinawa Island (sites 8–11), which has a subtropical climate, may be one of the major factors behind the increase in the number of pest species recorded, since Okinawa has insect species unique to the region, such as spotted grasshoppers, tussock caterpillars, Chinese thrips (*Haplothrips chinensis* Priesner, 1933), and *Prolygus* plant bugs (*Prolygus bakeri* (Poppius, 1915)).

Among the 38 species detected in the present study, onion thrips (*Thrips tabaci* Lindeman, 1889), two-spotted spider mites, and cotton whiteflies were collected from more than half of the study sites, that is, from 8 sites, 7 sites, and 6 sites, respectively. Moreover, these three species, on an empirical basis, were much more abundant on pepino plants than the other pest species, and from several hundred to thousands of individuals of these three species were found on each pepino plant (Fig. 15). In Japan, these three species may be considered the most important insect and mite pests of pepino plants.

In the world, 25 species of insects and mites are known as pests of pepino plants and seven species of them are considered as important pests (Larraín 2002; Galbreath and Clearwater 1983; Akyazi 2012). Of these seven, four species, namely two-spotted spider mites, green peach aphids, *solenopsis* mealybugs (*Phenacoccus solenopsis* Tinsley, 1898), and broad mites, are distributed in Japan. The former two species are common to Japan and the world as pests of pepino plants. The latter two species have not been found so far from pepino plants in Japan, but attention should be paid to future trends. On the other hand, onion thrips and tobacco whiteflies, which are considered to be likely the most important pests in Japan in the present study, are not important in other countries to date; however, these two species might be important pests because they are distributed worldwide.

Although most of the Japanese pest species of pepino plants are leaf-feeders, two lepidopteran species, tussock caterpillars and tobacco budworms (*Helicoverpa armigera* (Hübner, 1808)), were observed feeding on the fruits of pepino plants in the current study (Fig. 16). This results in holes in the fruits, which may negatively affect the commercial value of pepino. Pest management will be important for the cultivation of pepino plants, because no pesticides applicable to these plants have been registered in Japan to date. Therefore, biological control will have to be used for the commercial cultivation of pepino at the moment.

## Supplementary Material

45613735-ef88-5044-aa15-1a0a3ede4813 10.3897/BDJ.7.e36453.suppl1 Supplementary material 1 PEPINO_PESTS data Data type: occurrences
Brief description: Occurrences of insect and mite species of pests of pepino plants in Japan.
File: oo_321746.xls https://binary.pensoft.net/file/321746 T. Ishikawa, K. Takahata

## Figures and Tables

**Figure 2a.**
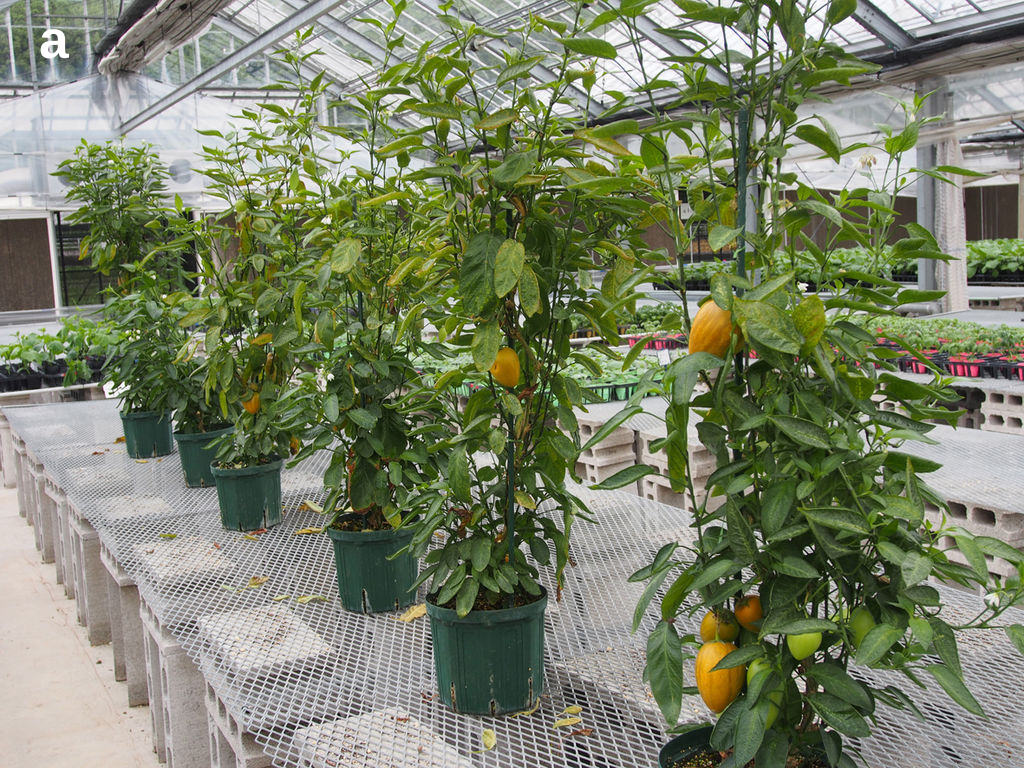
Study site 1, the inside of a greenhouse in Tochigi Prefecture (36.439N 139.668E).

**Figure 2b.**
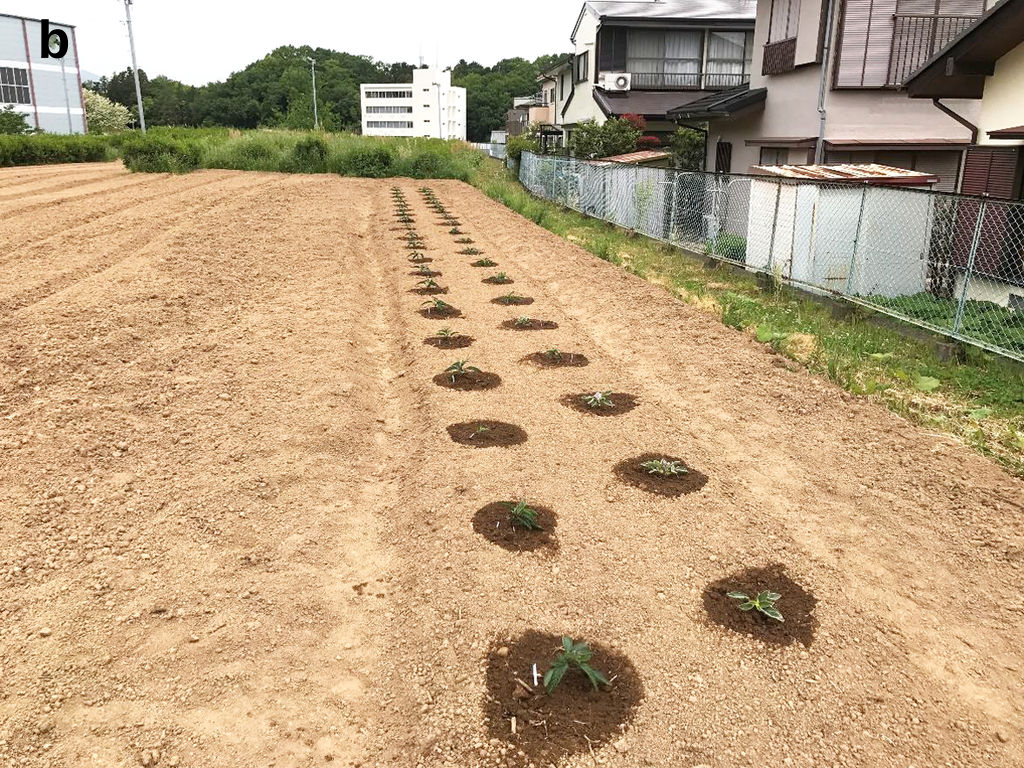
Study site 2, an open field in Kanagawa Prefecture (35.433N 139.348E), just after planting of nursery pepinos.

**Figure 2c.**
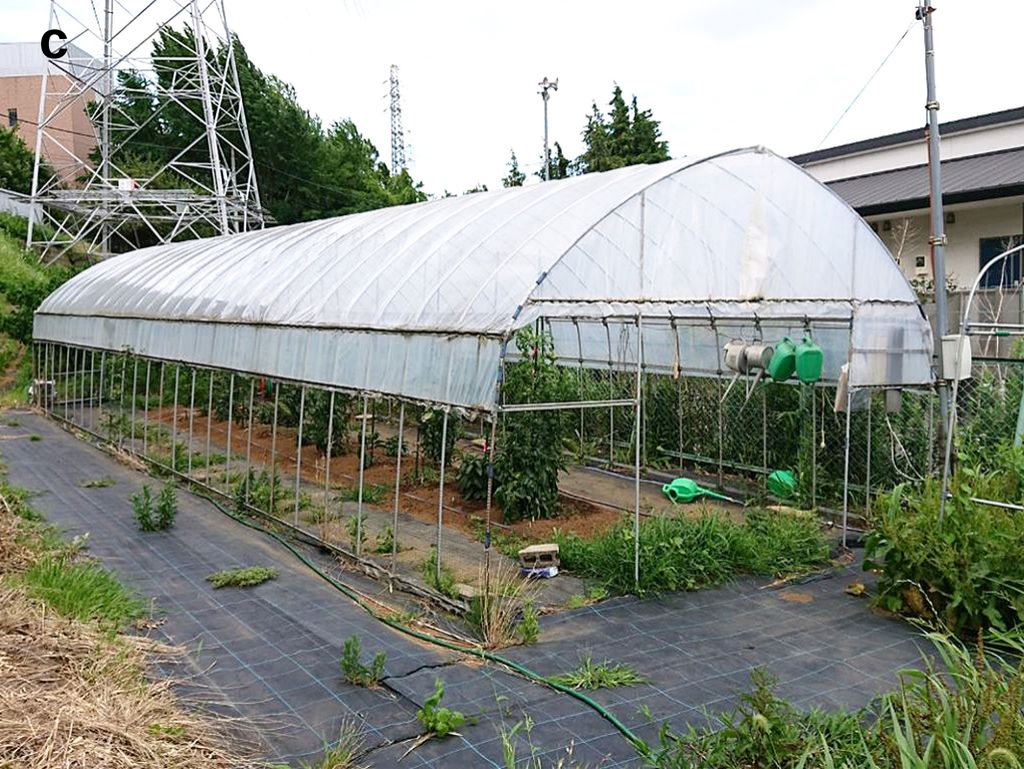
Study site 3, an open field (with a roof against rain) in Kanagawa Prefecture (35.432N 139.346E).

**Figure 2d.**
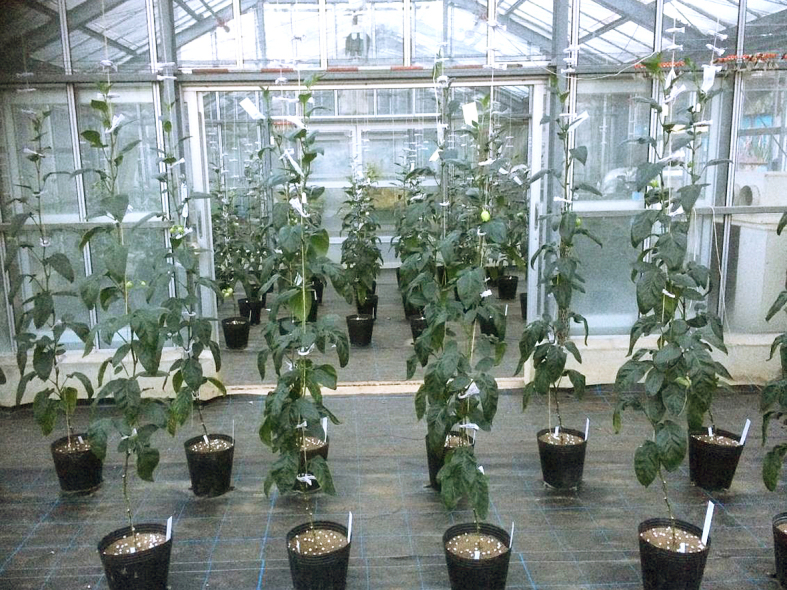
Study site 4, the inside of a greenhouse in Kanagawa Prefecture (35.431N 139.350E).

**Figure 2e.**
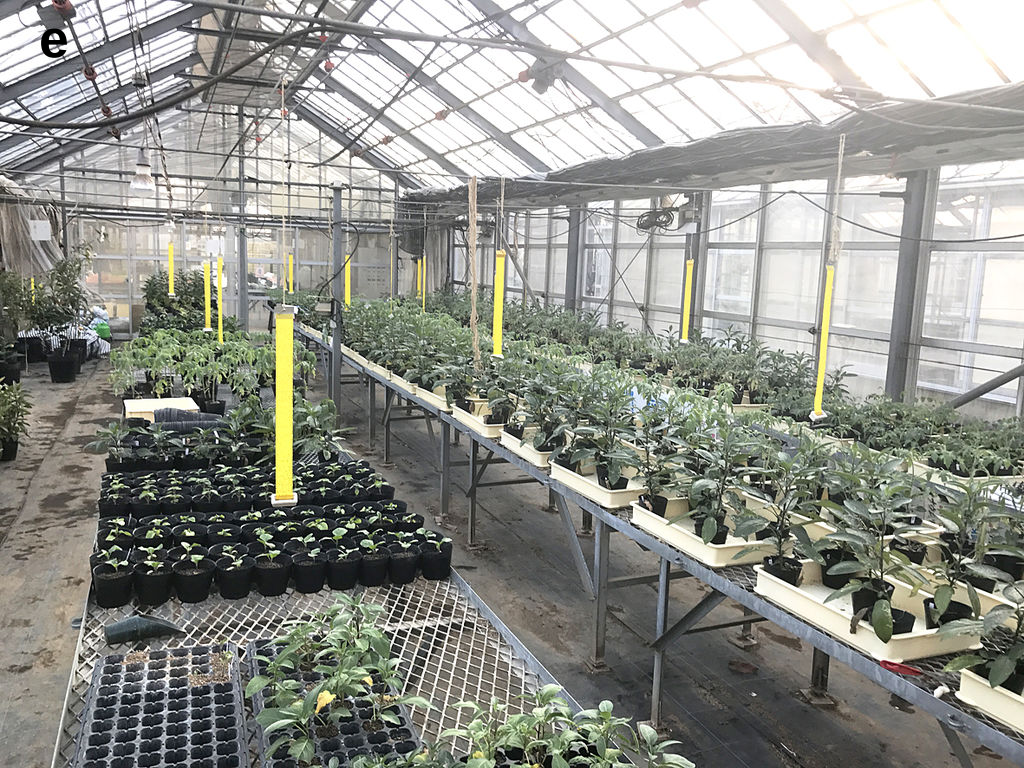
Study site 5, the inside of a greenhouse in Kanagawa Prefecture (35.429N 139.349E).

**Figure 2f.**
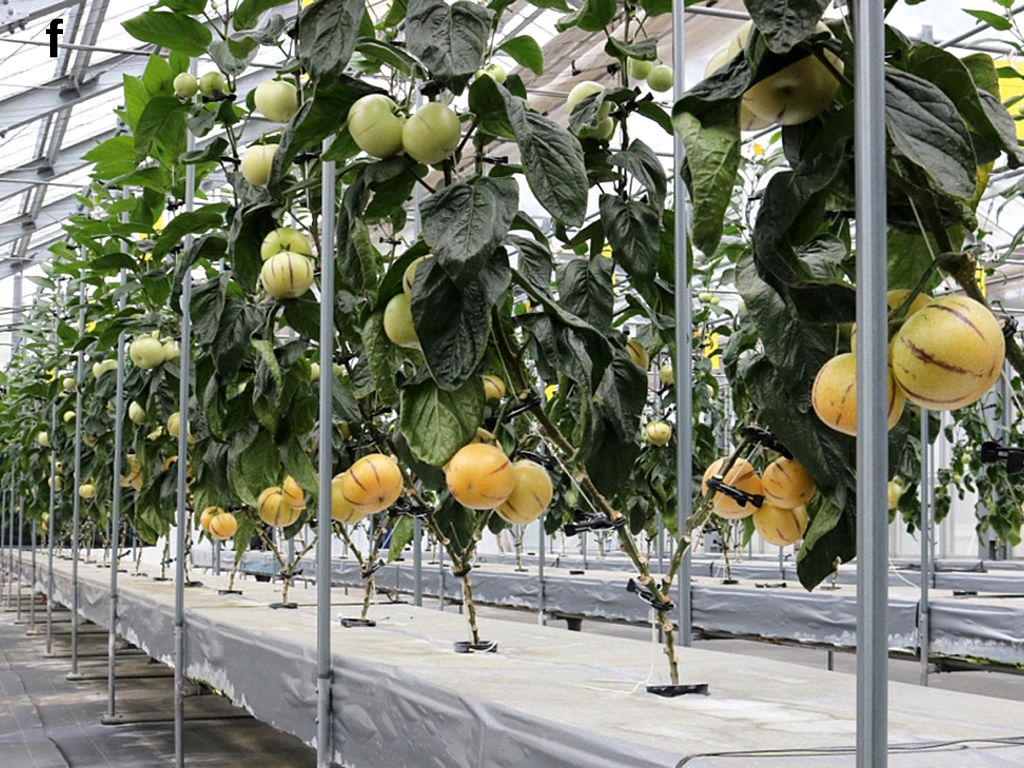
Study site 6, the inside of a greenhouse in Kanagawa Prefecture (35.400N 139.282E).

**Figure 3a.**
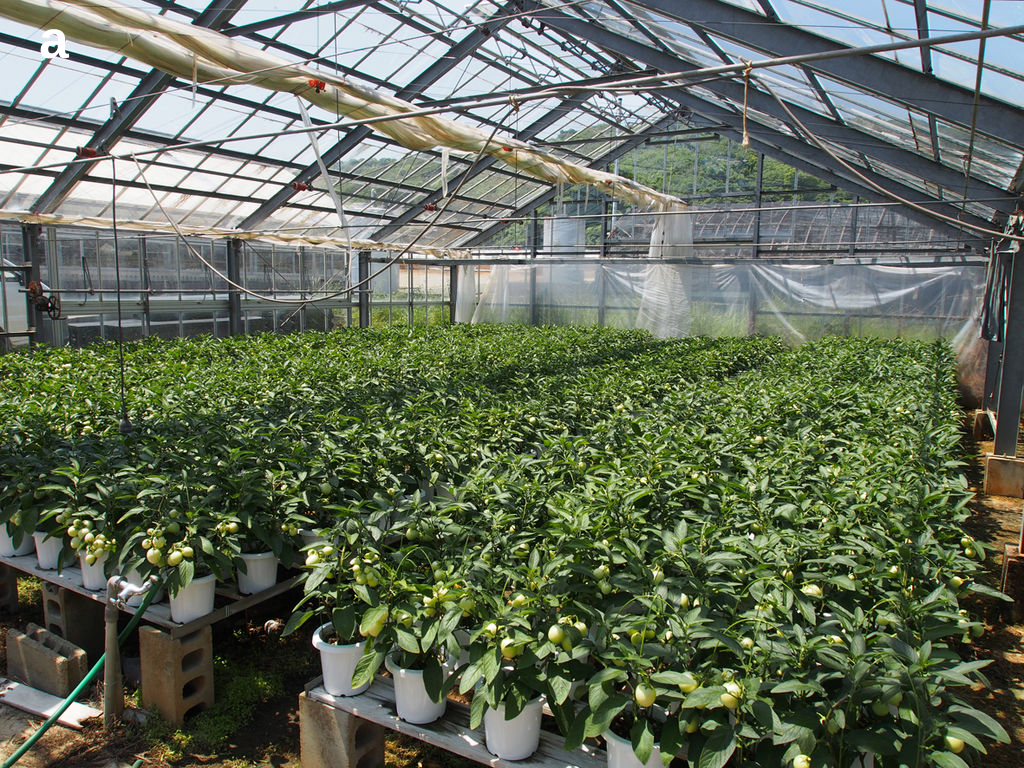
Study site 7, the inside of a greenhouse in Aichi Prefecture (34.600N 137.097E).

**Figure 3b.**
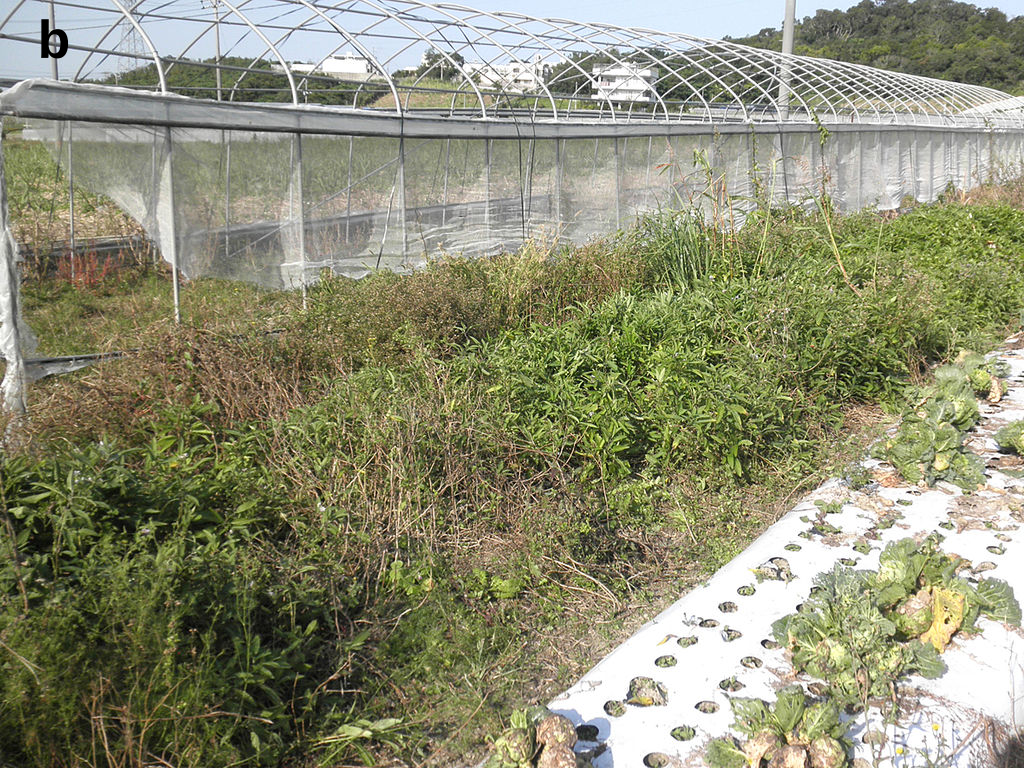
Study site 8, an open field in Okinawa Prefecture (26.189N 127.735E).

**Figure 3c.**
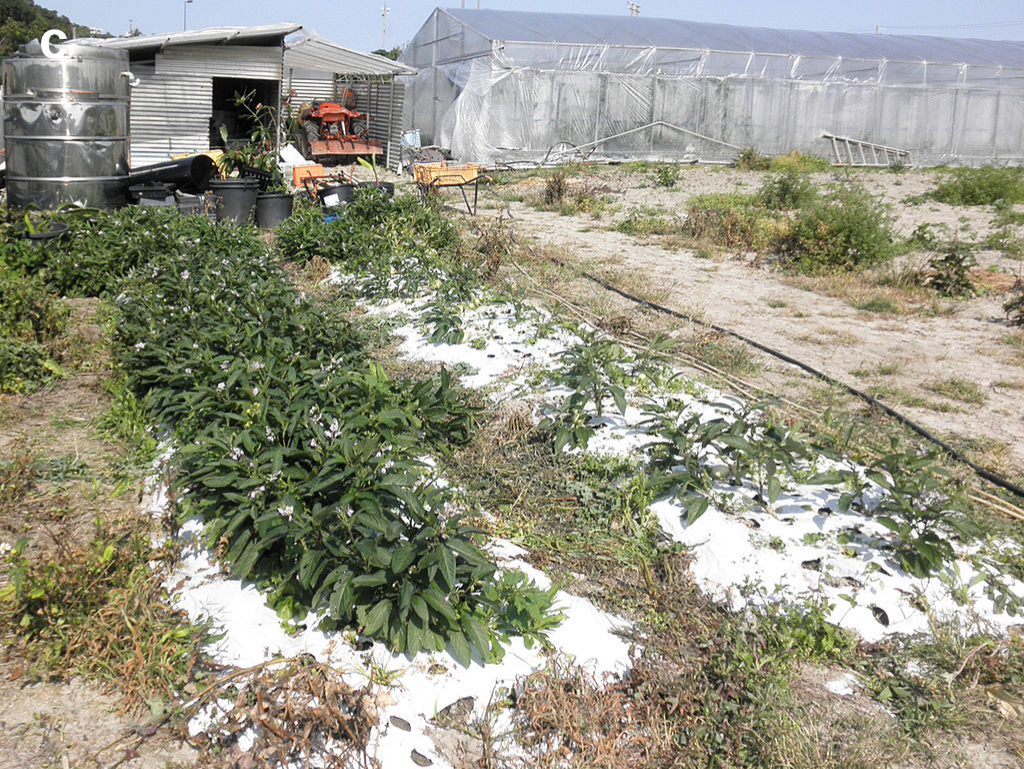
Study site 9, an open field in Okinawa Prefecture (26.186N 127.736E).

**Figure 3d.**
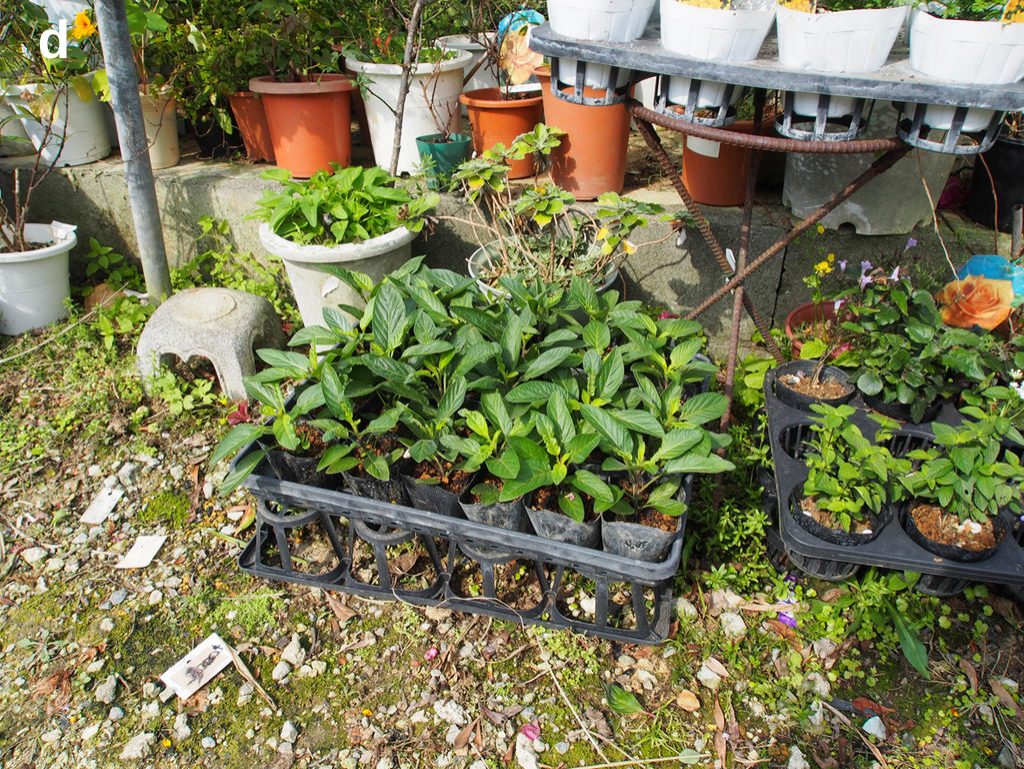
Study site 10, a garden center in Okinawa Prefecture (26.172N 127.734E), pepino nursery stocks (shown in the middle) are lined up with other plant pots.

**Figure 3e.**
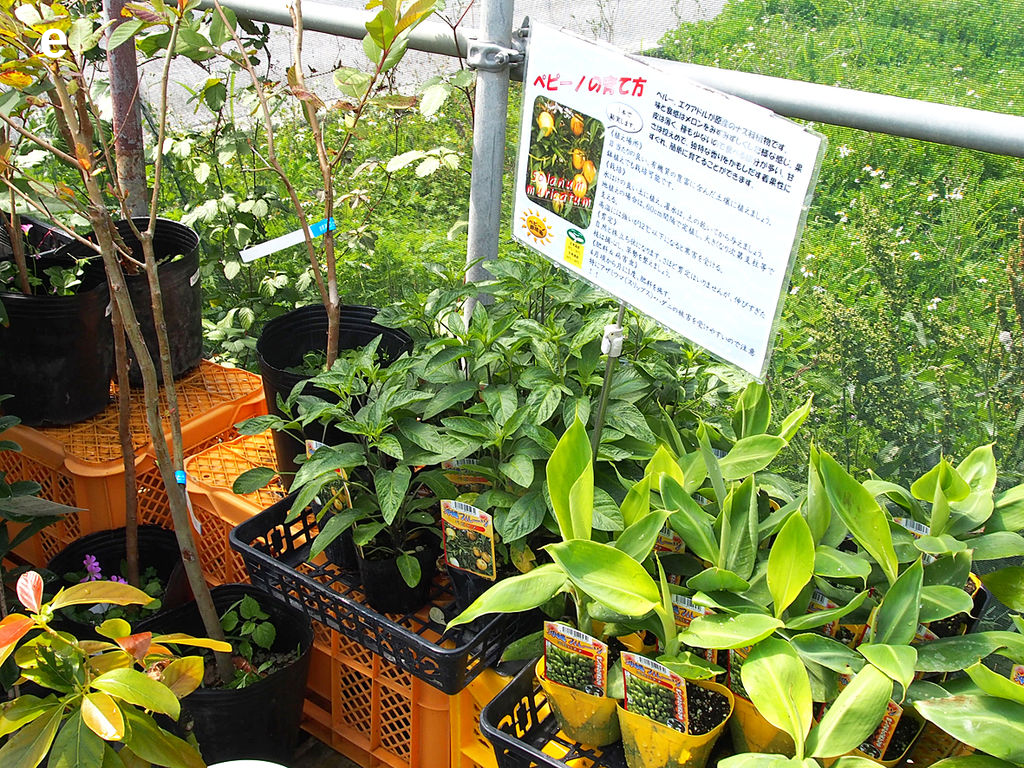
Study site 11, a garden center in Okinawa Prefecture (26.171N 127.737E), pepino nursery stocks (shown in the middle) are lined up with other plant pots.

**Figure 4.**
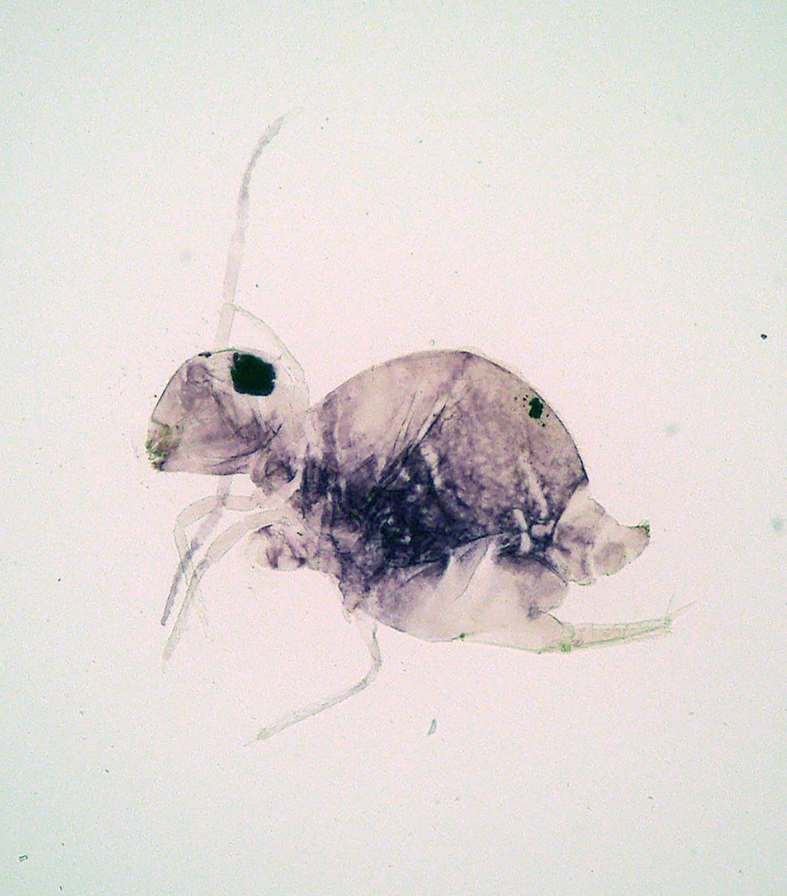
A garden springtail (*Bourletiella hortensis*, Bourletiellidae) feeding on pepino.

**Figure 5.**
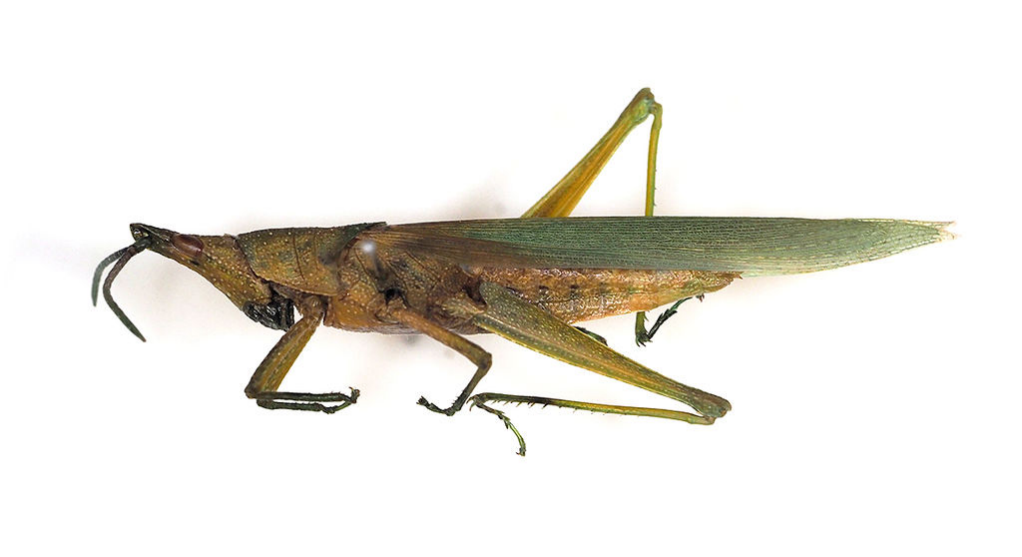
A spotted grasshopper (*Atractomorpha sinensis*, Pyrgomorphidae) feeding on pepino.

**Figure 6a.**
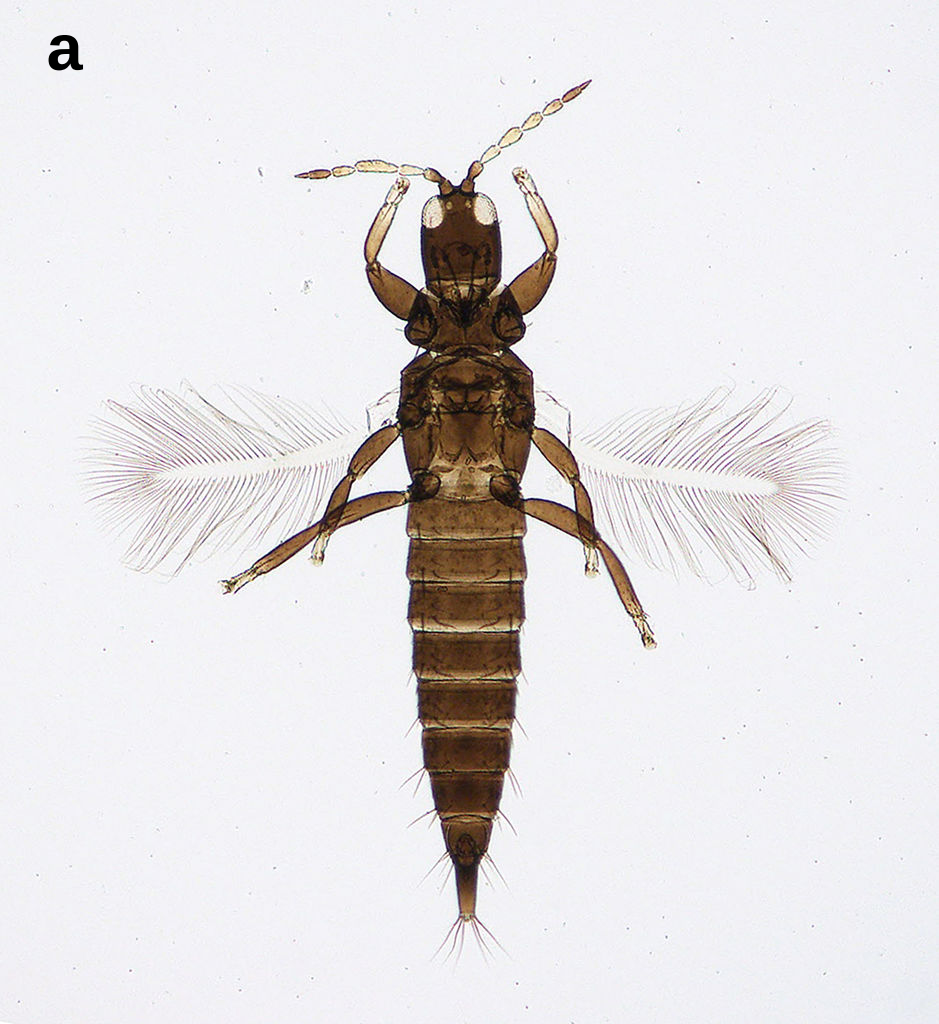
a Chinese thrips (*Haplothrips chinensis*, Phlaeothripidae).

**Figure 6b.**
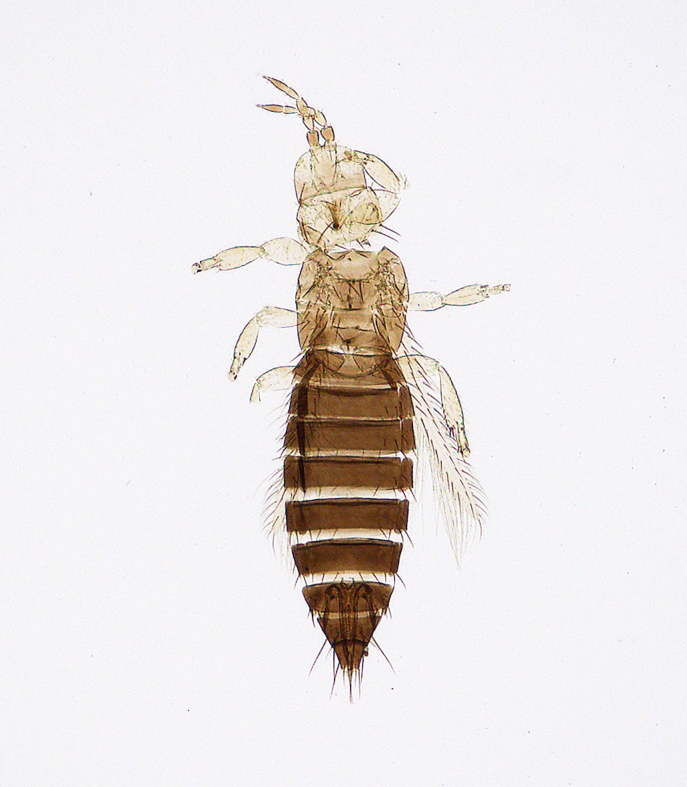
a flower thrips (*Frankliniella intonsa*, Thripidae).

**Figure 6c.**
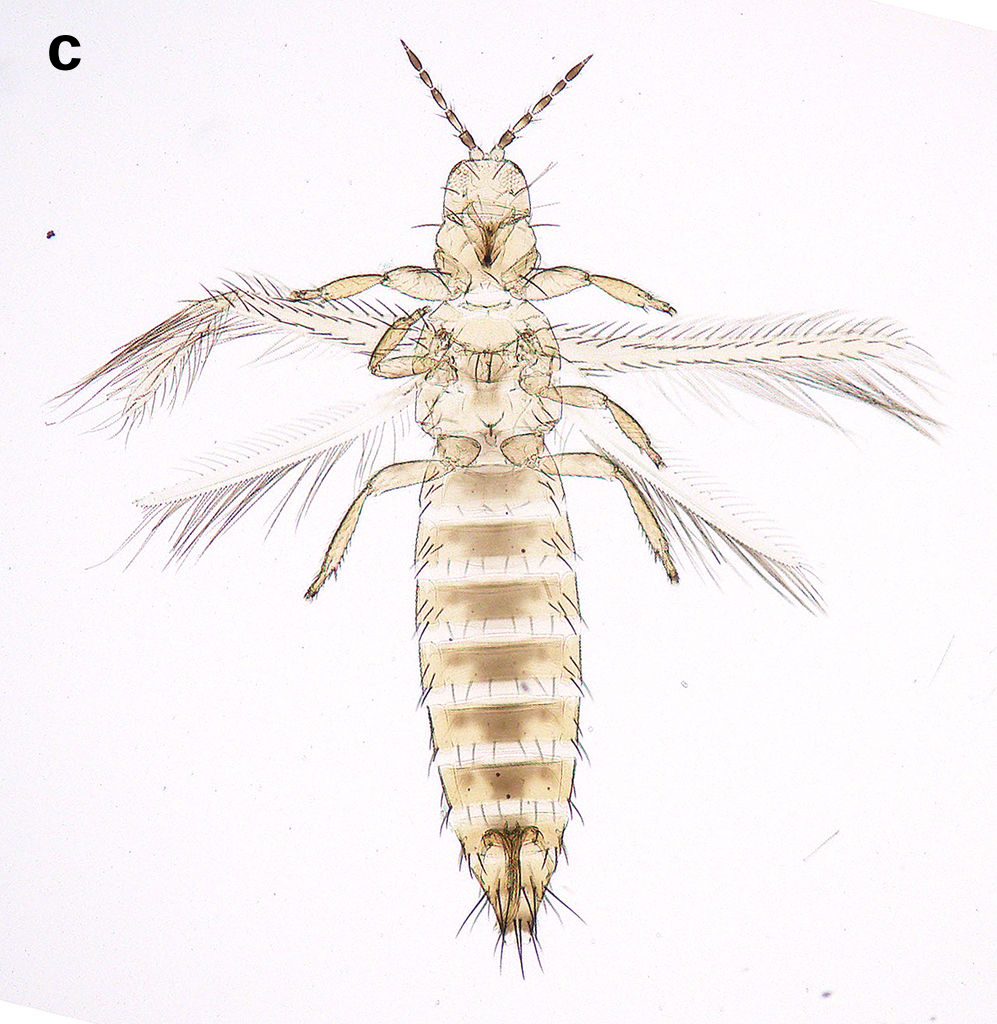
a western flower thrips (*Frankliniella occidentalis*, Thripidae).

**Figure 6d.**
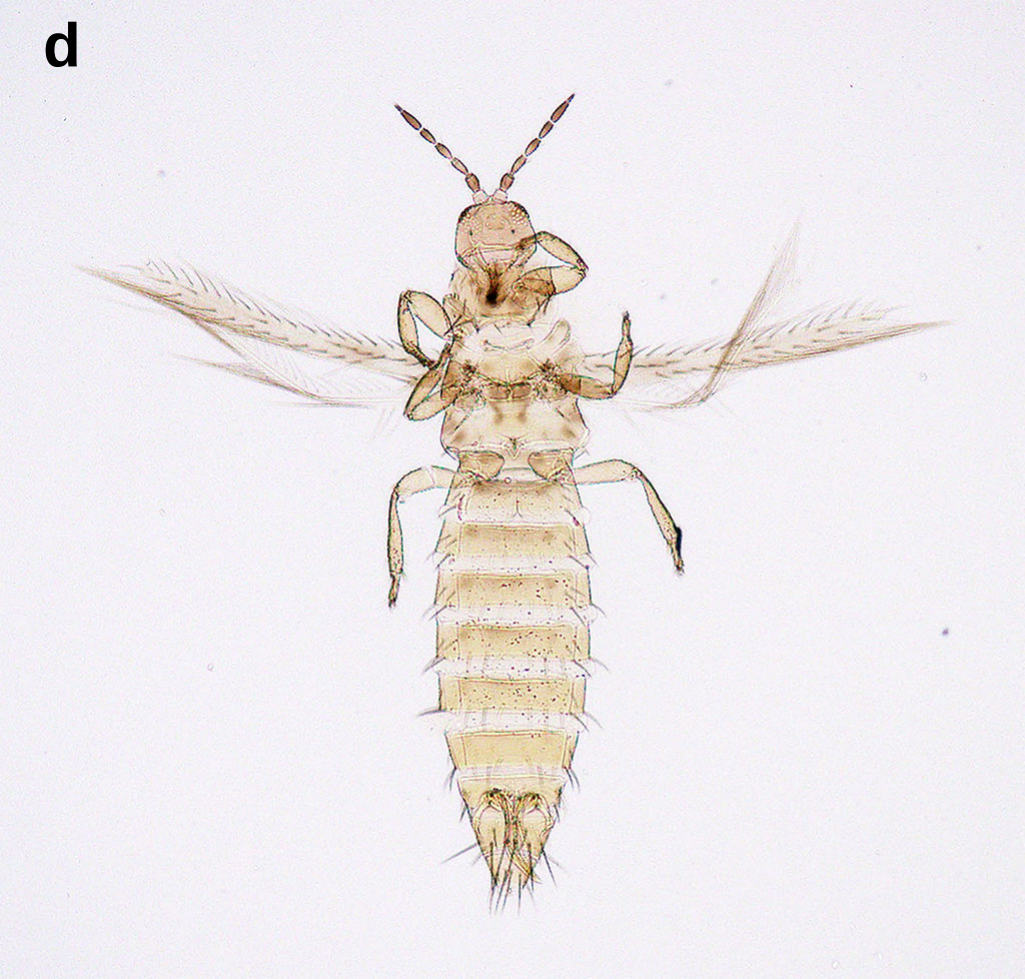
a chrysanthemum thrips (*Thrips nigropilosus*, Thripidae).

**Figure 6e.**
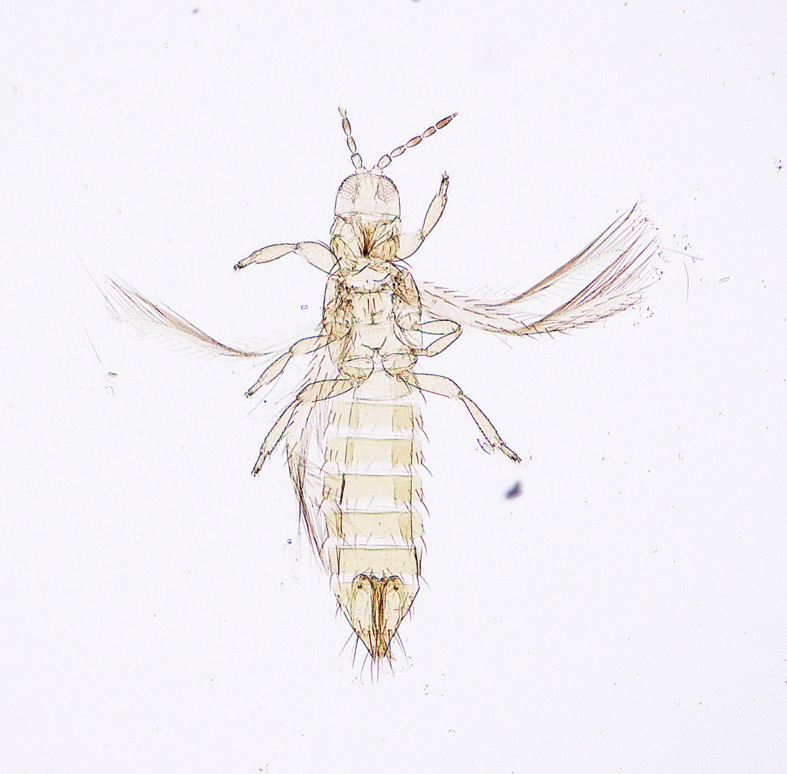
a melon thrips (*Thrips palmi*, Thripidae).

**Figure 6f.**
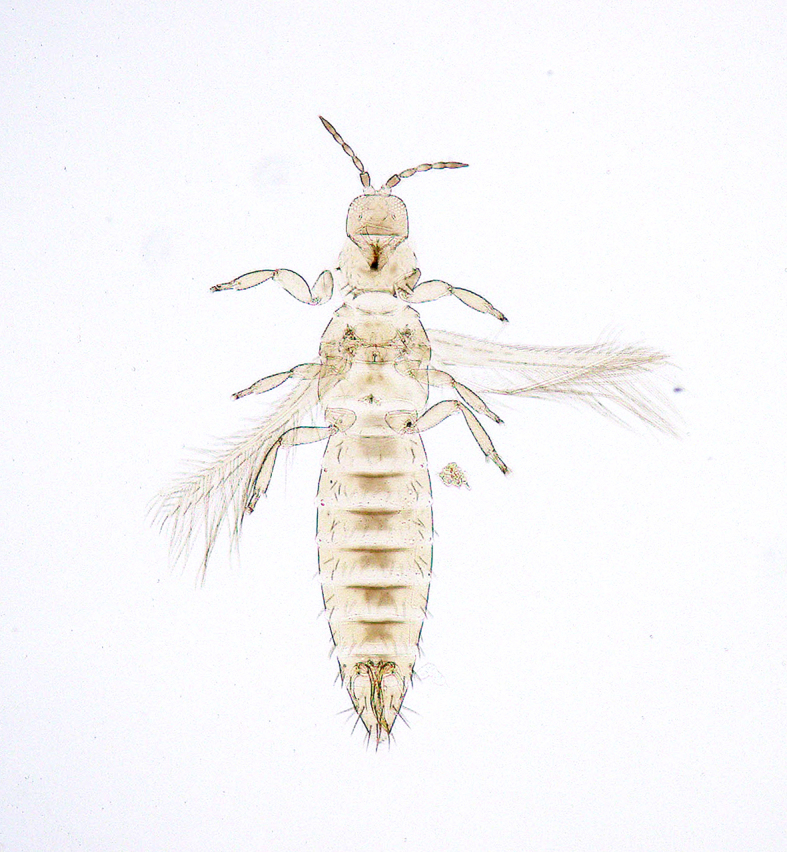
an onion thrips (*Thrips tabaci*, Thripidae).

**Figure 7a.**
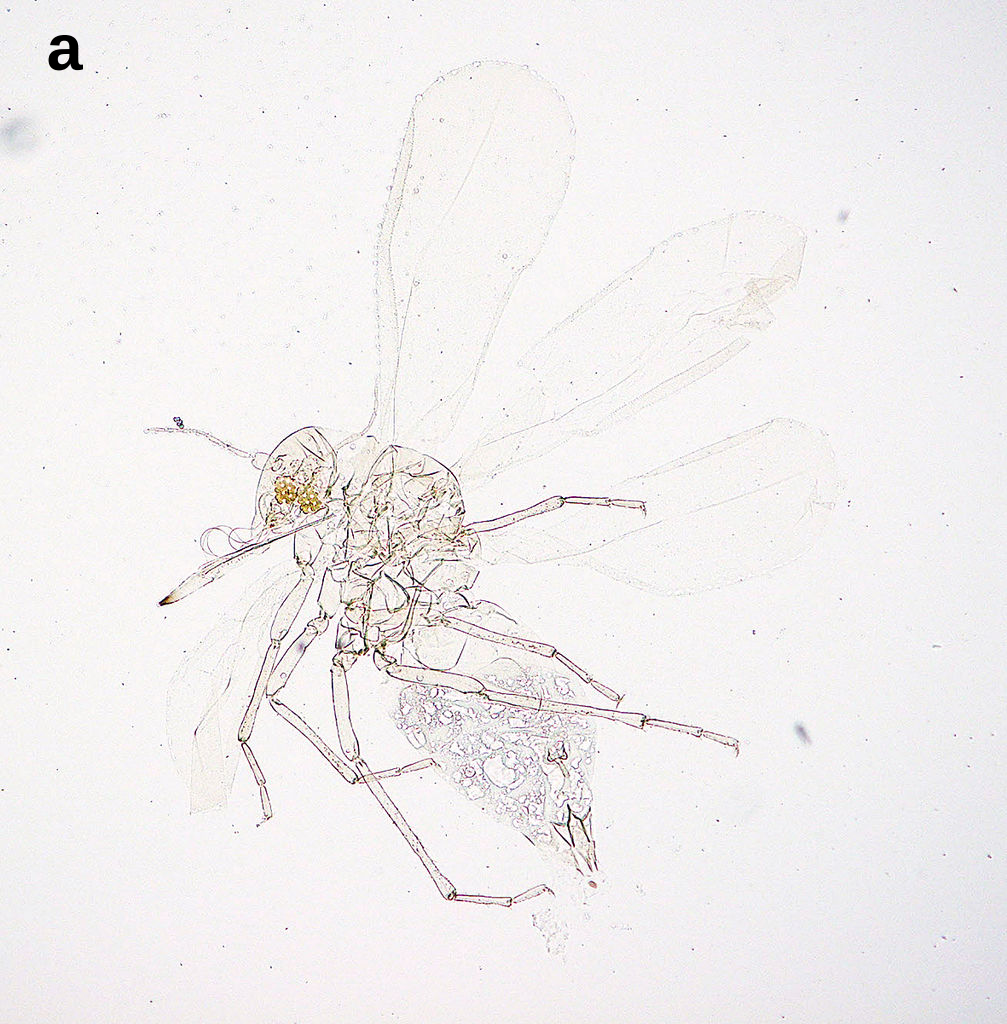
a cotton whitefly (*Bemisia tabaci*, Aleyrodidae).

**Figure 7b.**
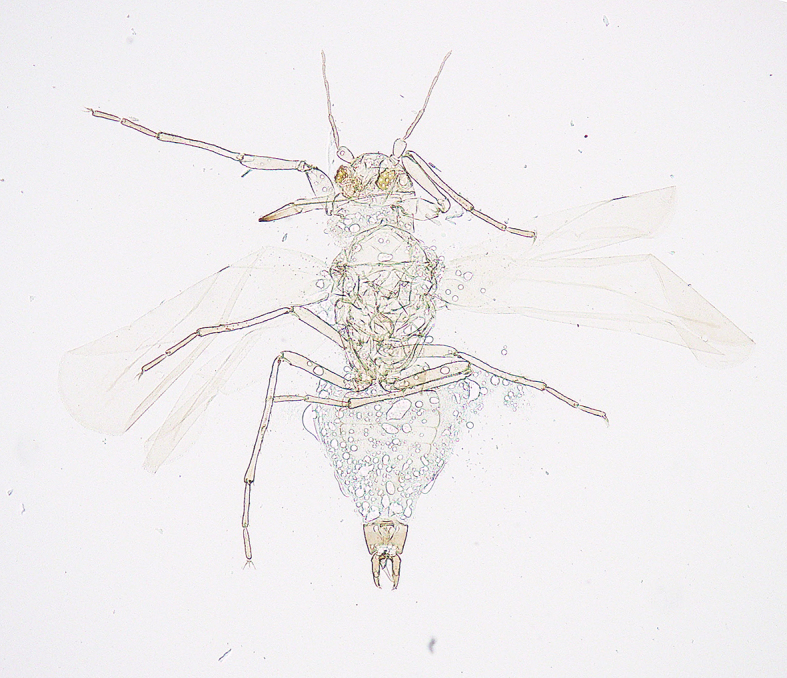
a greenhouse whitefly (*Trialeurodes vaporariorum*, Aleyrodidae).

**Figure 7c.**
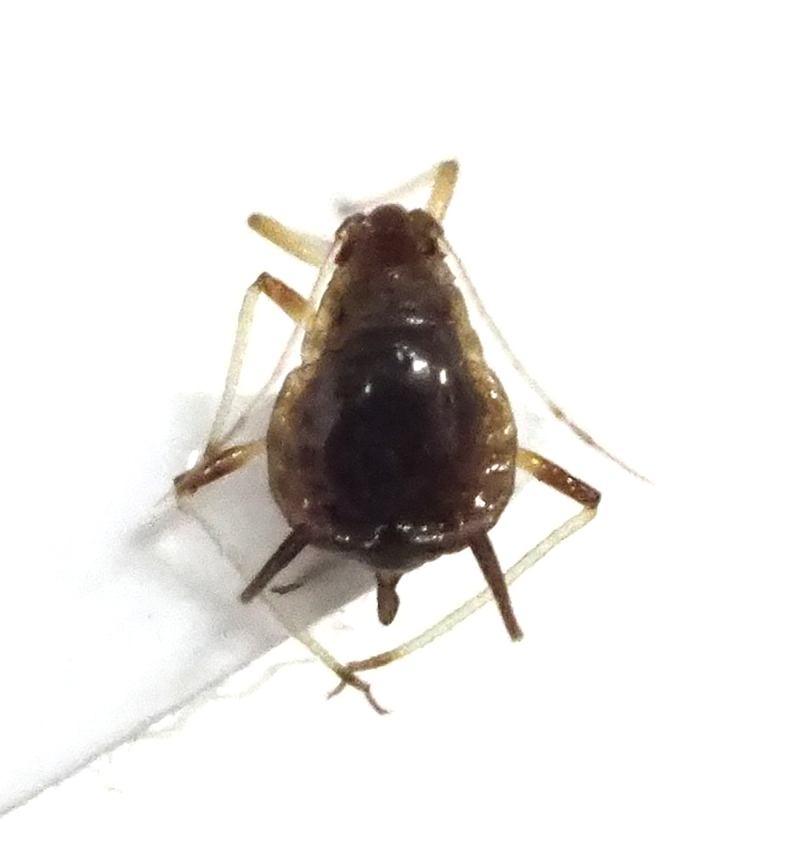
a cotton aphid (*Aphis gossypii*, Aphididae).

**Figure 7d.**
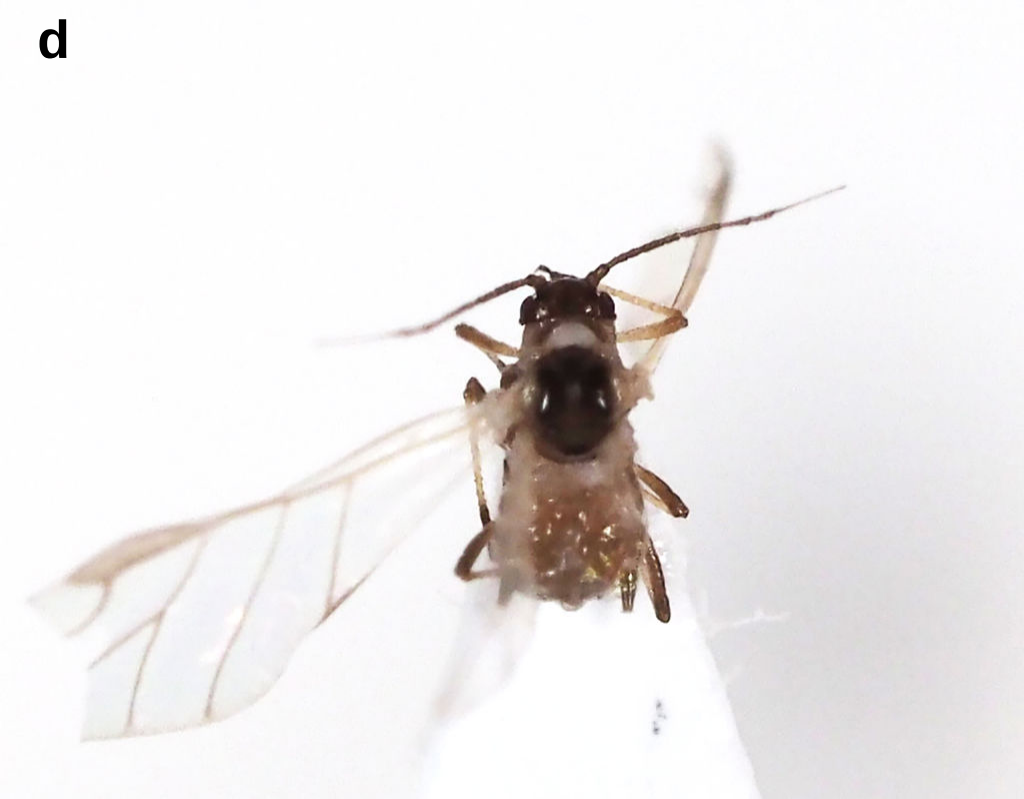
a Spiraea aphid (*Aphis spiraecola*, Aphididae).

**Figure 7e.**
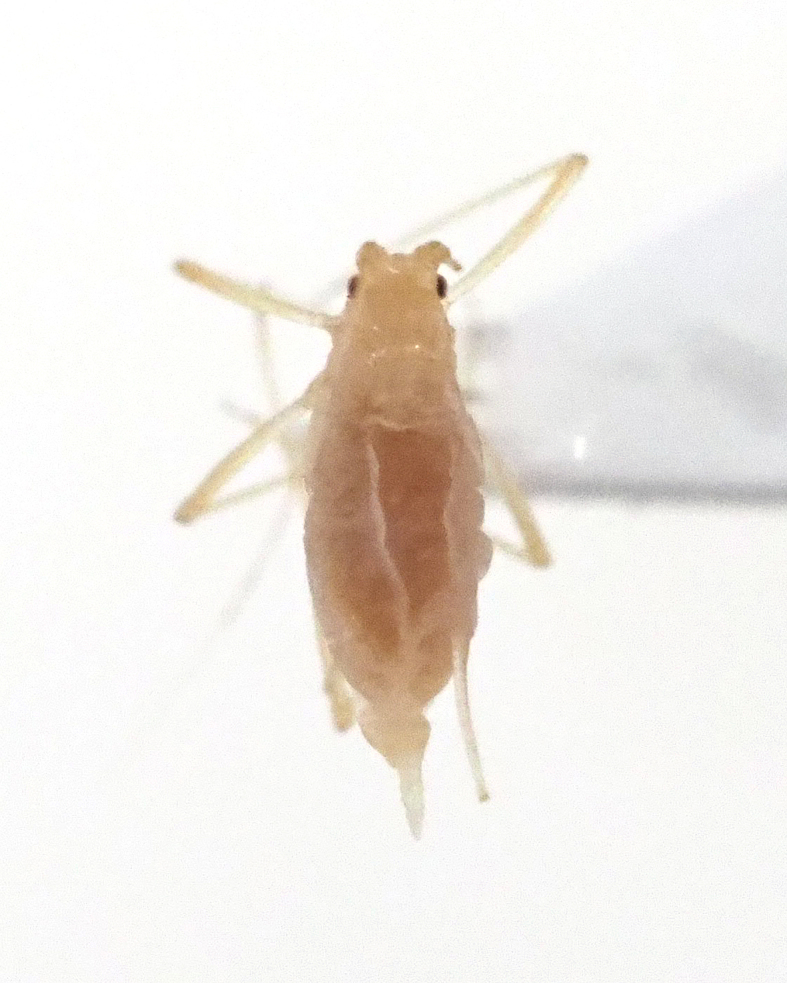
a potato aphid (*Macrosiphum euphorbiae*, Aphididae).

**Figure 7f.**
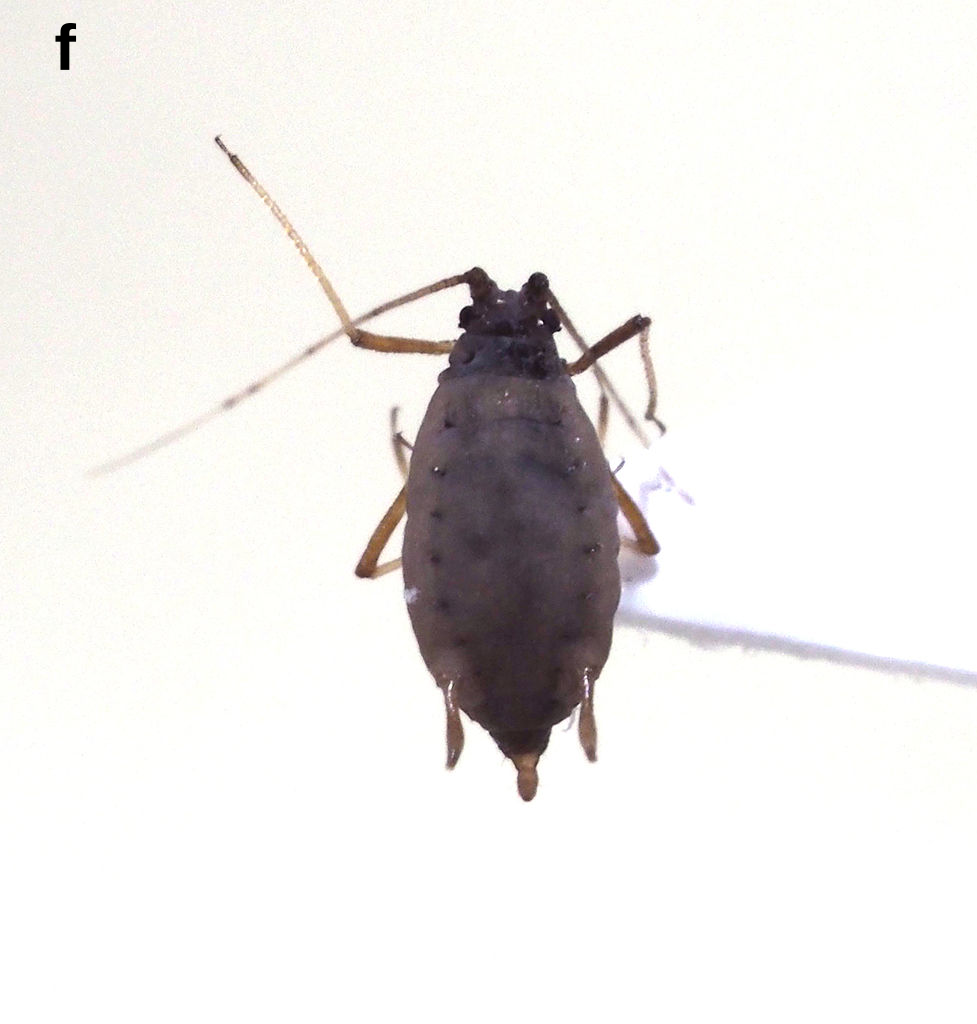
a green peach aphid (*Myzus persicae*, Aphididae).

**Figure 8.**
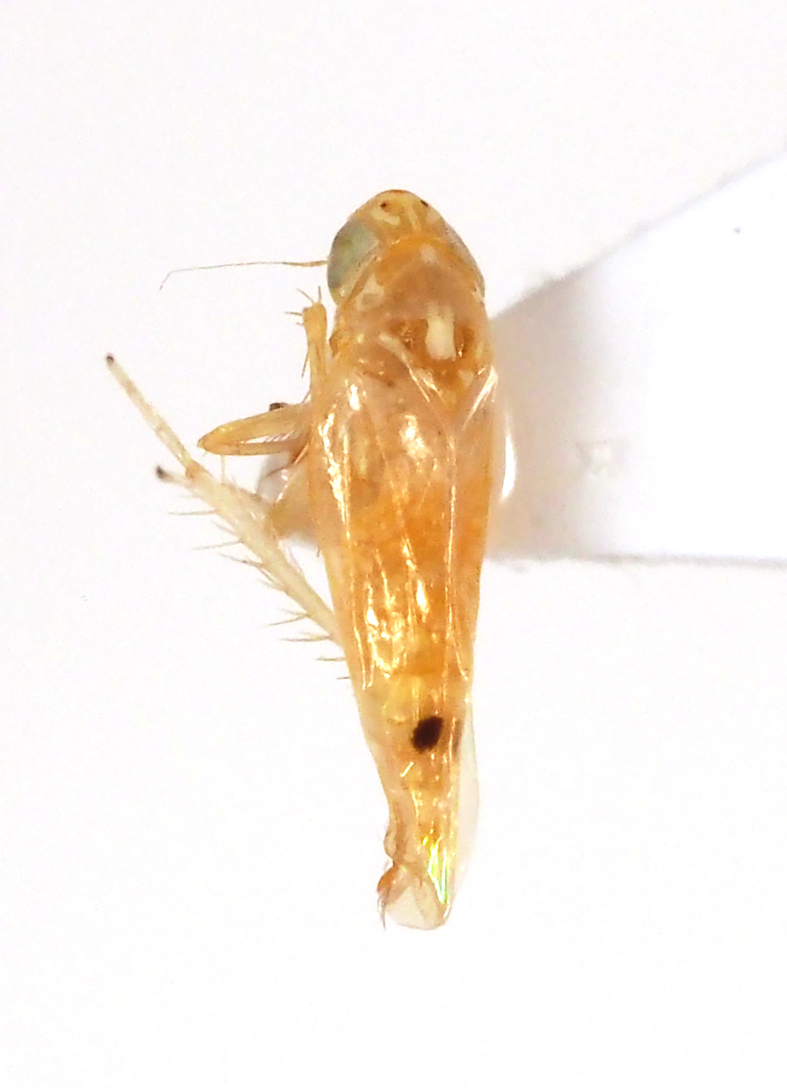
An *Amrasca* leafhopper (*Amrasca biguttula*, Cicadellidae) feeding on pepino.

**Figure 9a.**
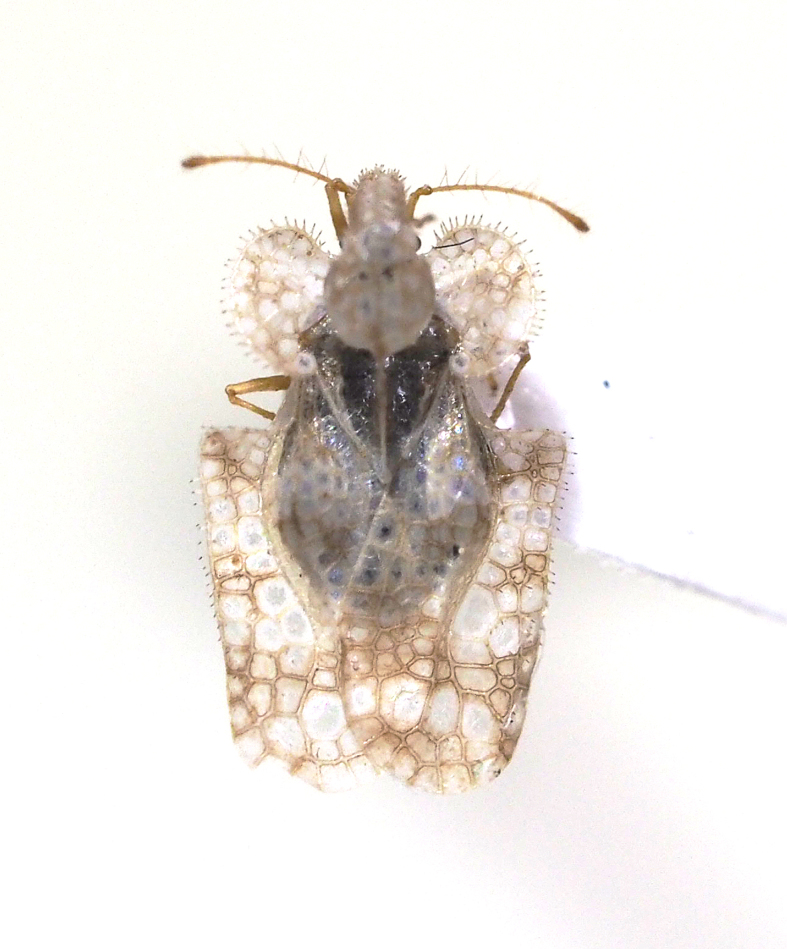
a chrysanthemum lace bug (*Corythucha marmorata*, Tingidae).

**Figure 9b.**
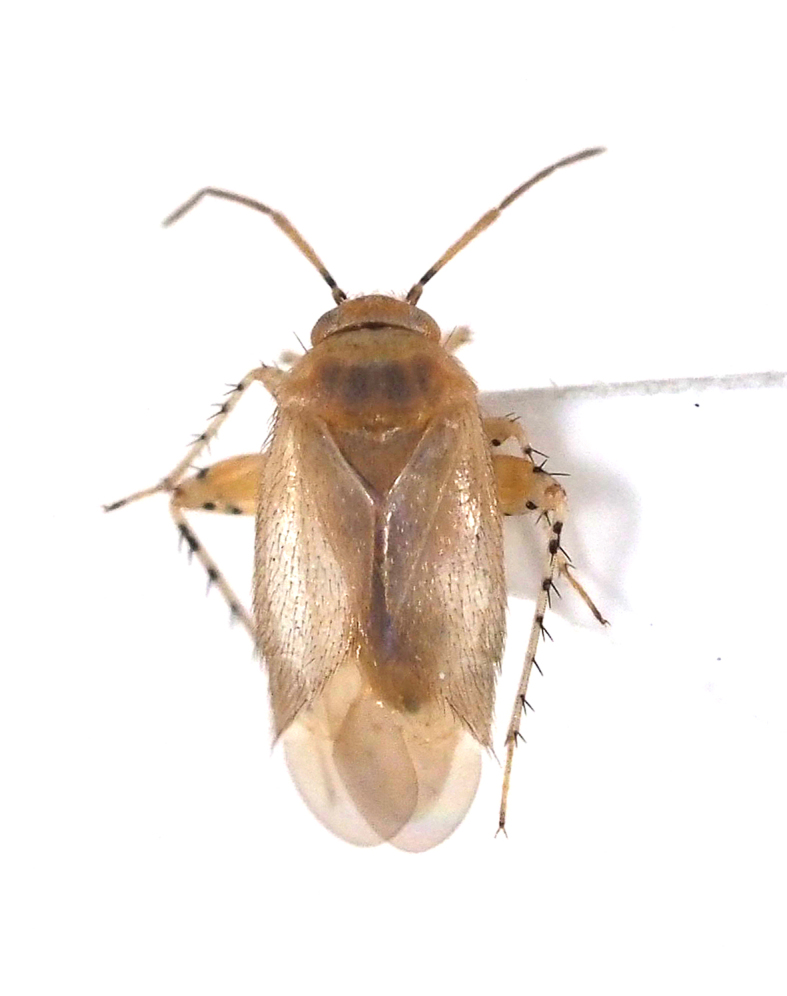
a *Campylomma* plant bug (*Campylomma livida*, Miridae).

**Figure 9c.**
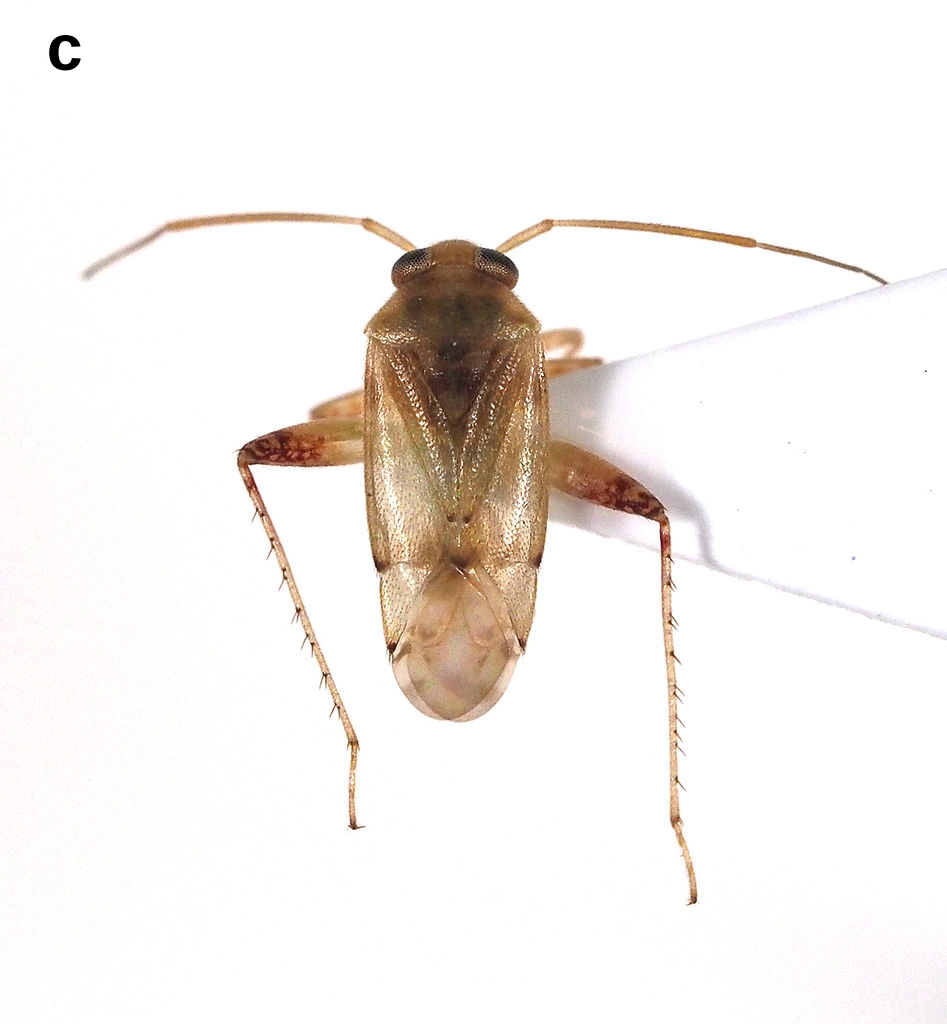
a *Prolygus* plant bug (*Prolygus bakeri*, Miridae).

**Figure 9d.**
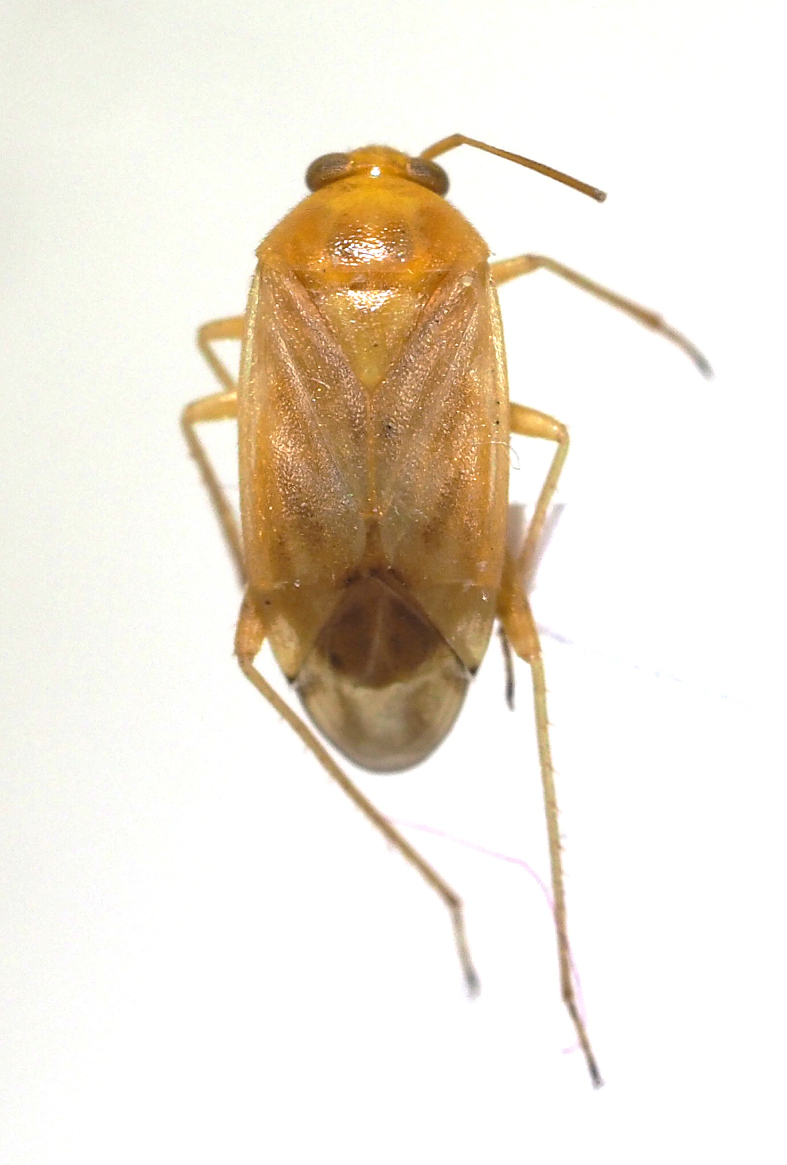
a *Taylorilygus* plant bug (*Taylorilygus apicalis*, Miridae).

**Figure 9e.**
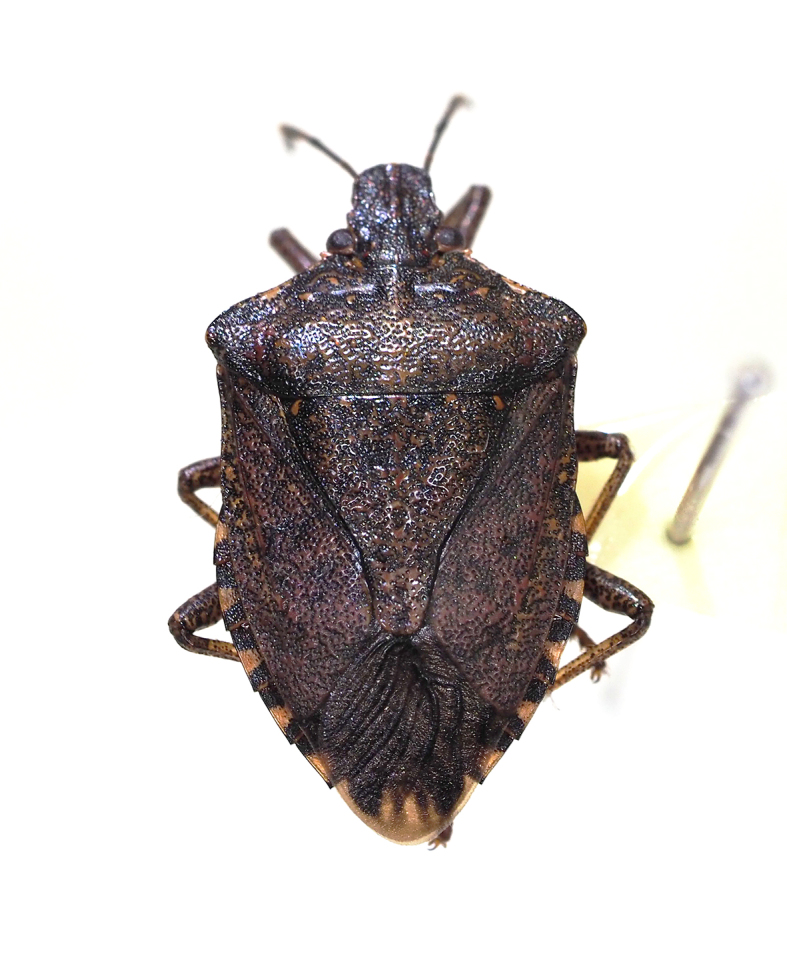
a brown marmorated stink bug (*Halyomorpha halys*, Pentatomidae).

**Figure 9f.**
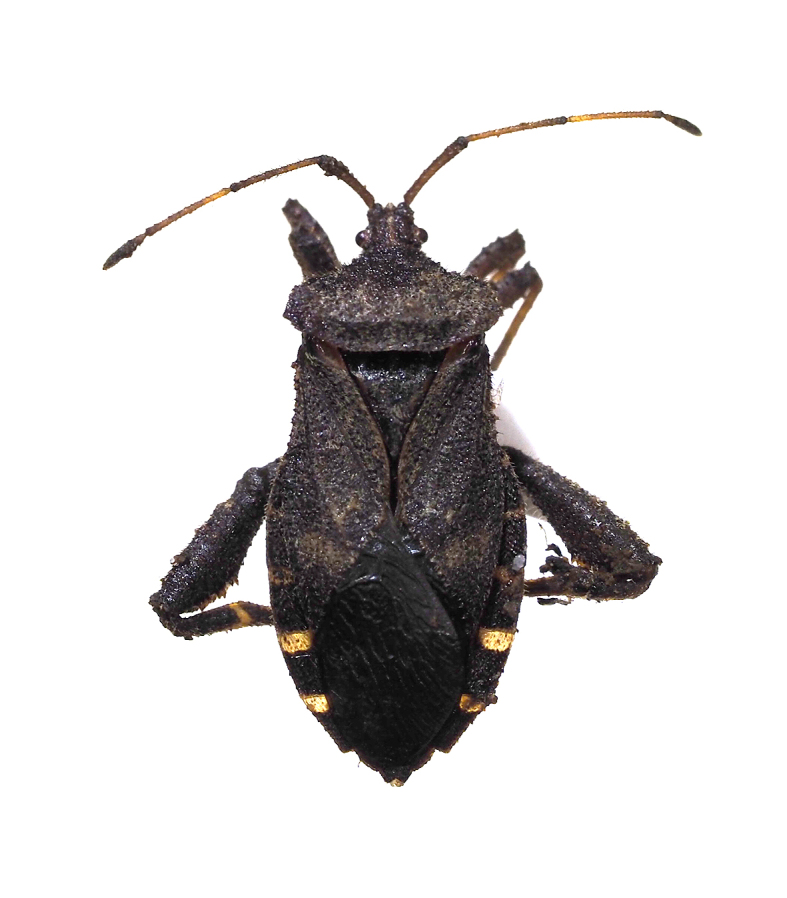
a winter cherry bug (*Acanthocoris sordidus*, Coreidae).

**Figure 10a.**
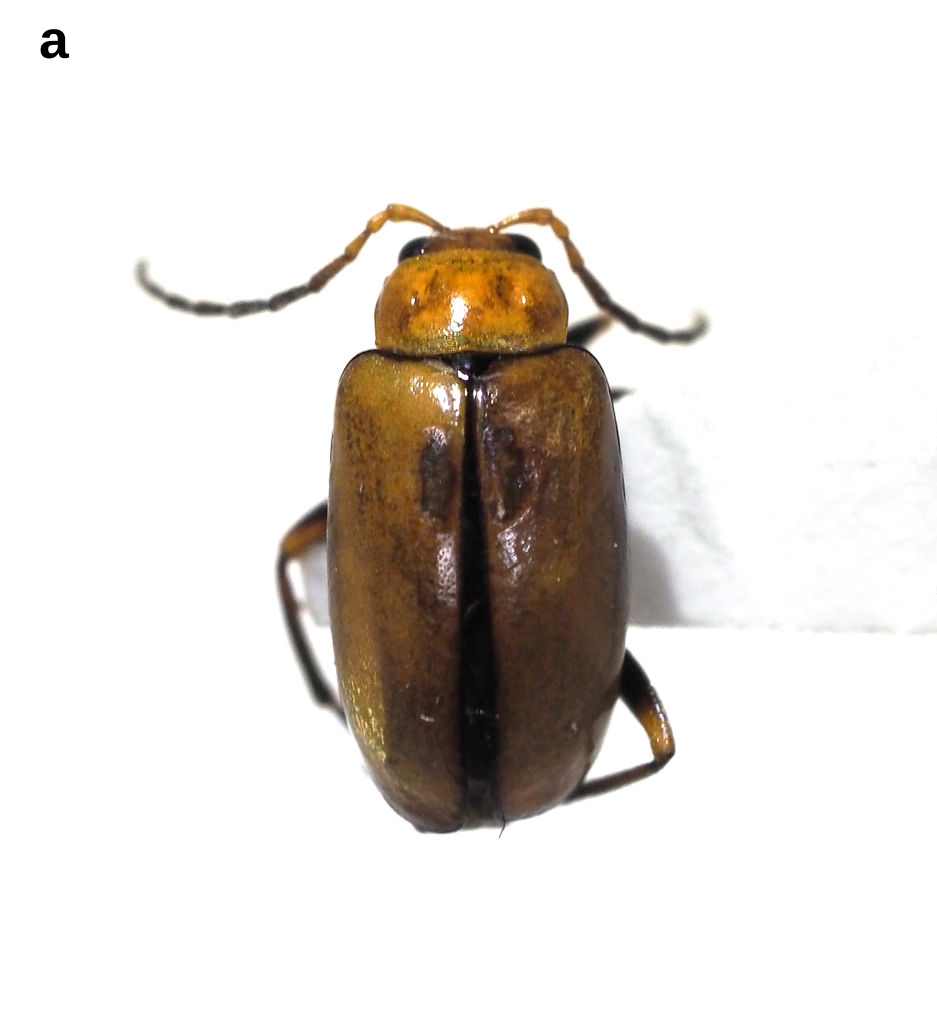
a false melon beetle (*Atrachya menetriesi*, Chrysomelidae).

**Figure 10b.**
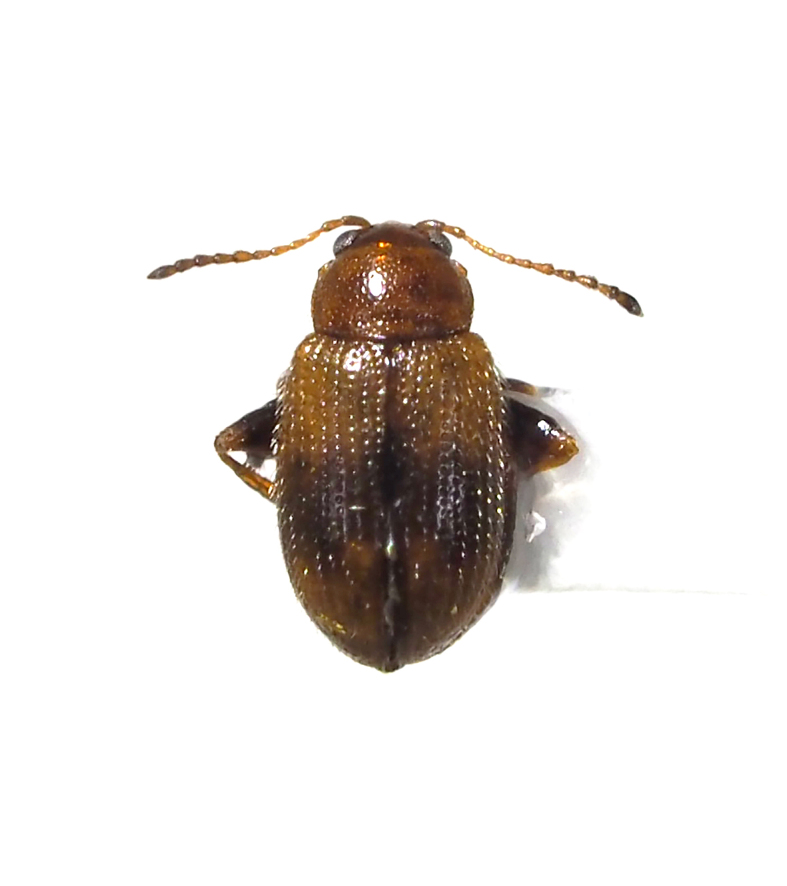
a tobacco flea beetle (*Epitrix hirtipennis*, Chrysomelidae).

**Figure 10c.**
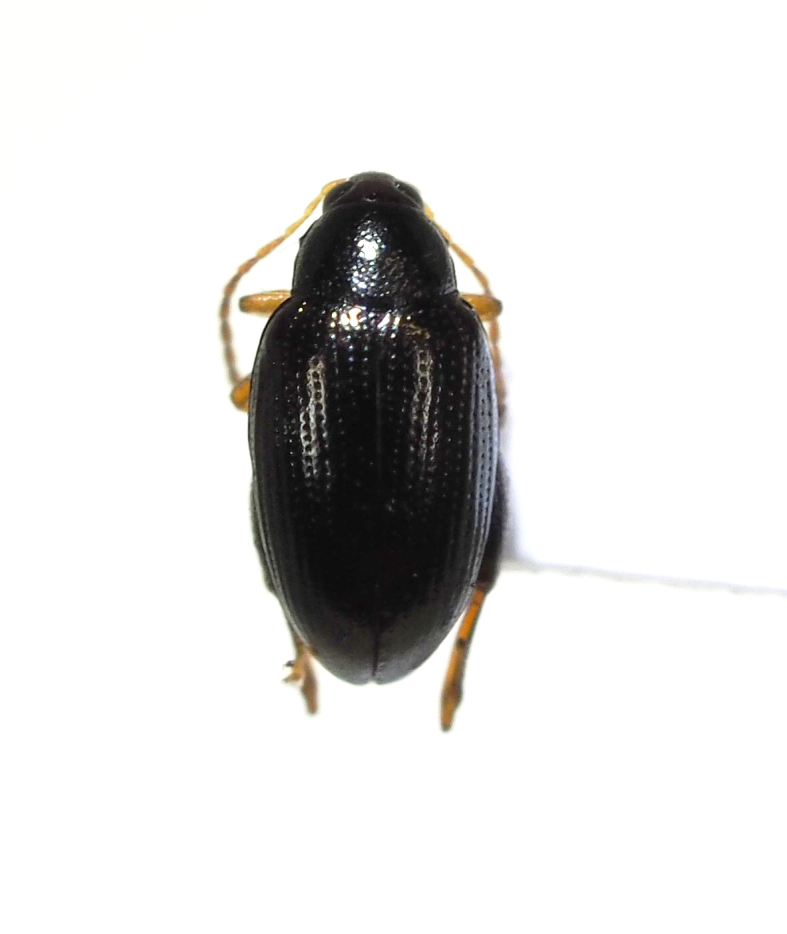
a solanum flea beetle (*Psylliodes angusticollis*, Chrysomelidae).

**Figure 10d.**
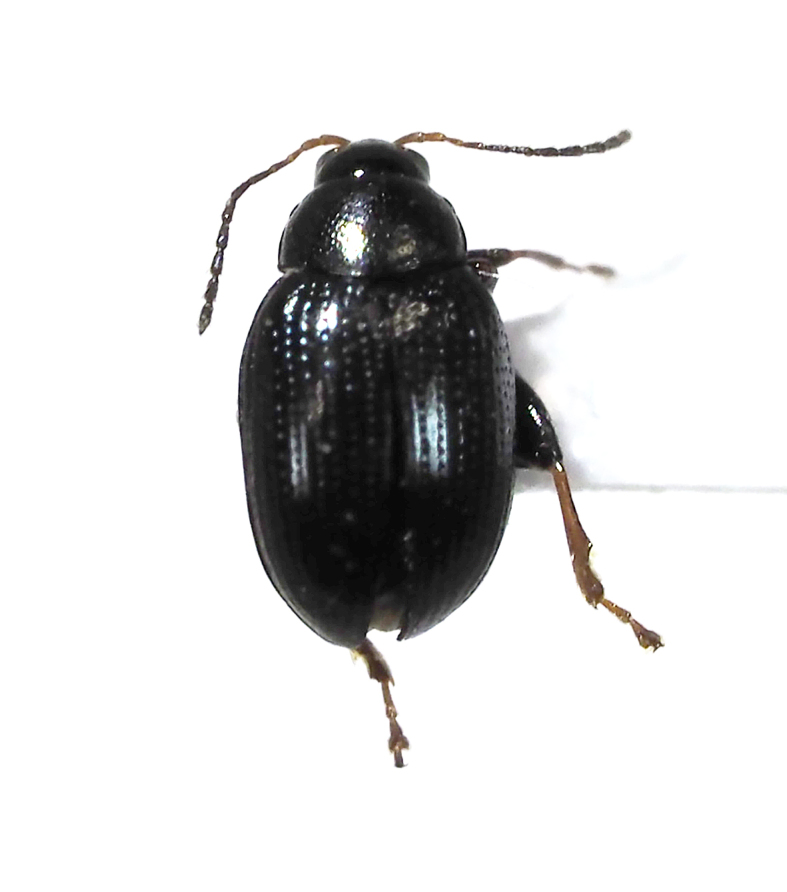
a cabbage flea beetle (*Psylliodes punctifrons*, Chrysomelidae).

**Figure 11a.**
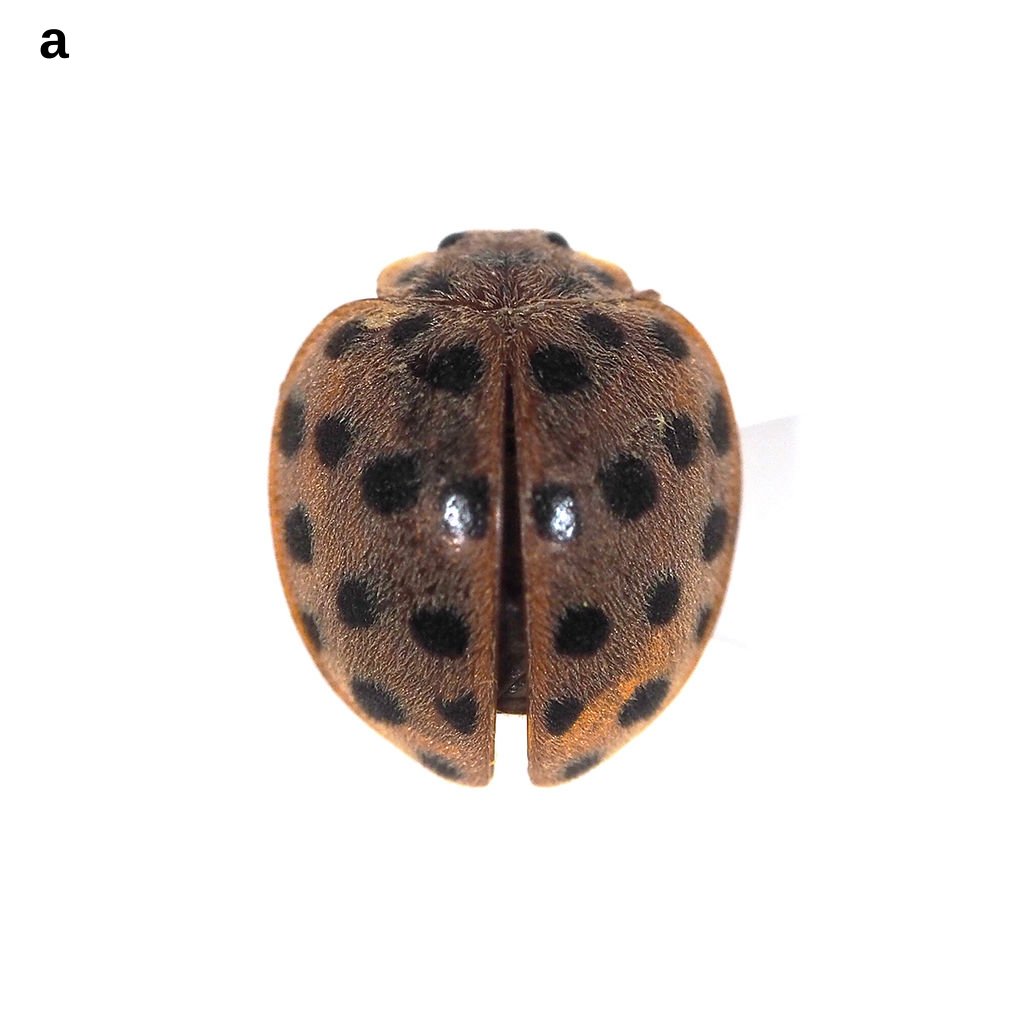
a twenty-eight-spotted ladybird (*Henosepilachna vigintioctopunctata*, Coccinellidae).

**Figure 11b.**
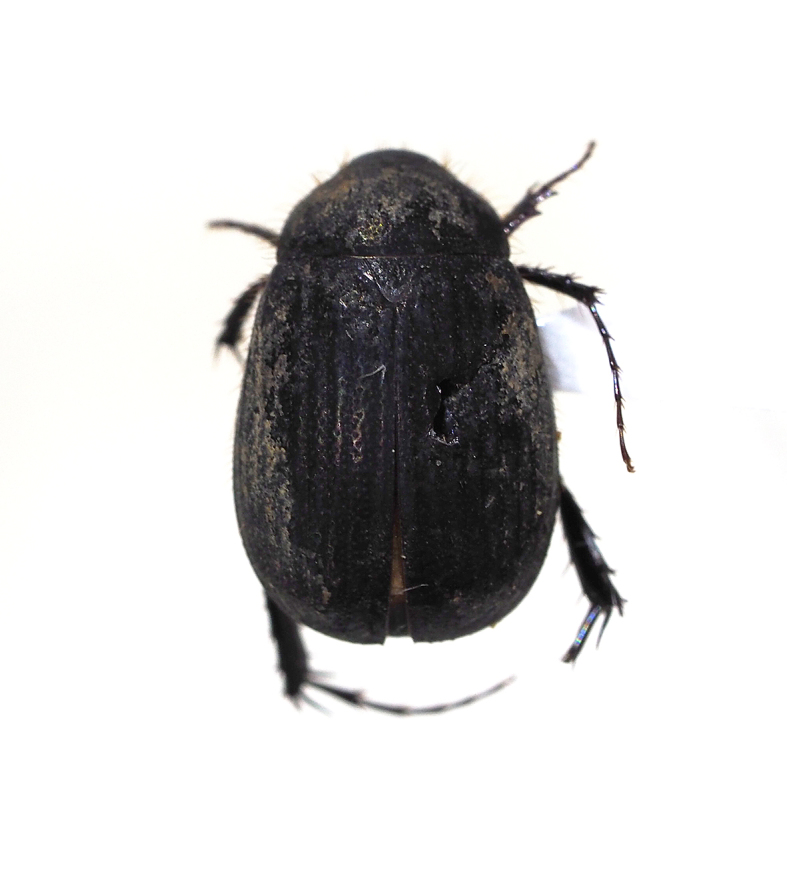
an *orientalis* garden beetle (*Maladera orientalis*, Scarabaeidae).

**Figure 11c.**
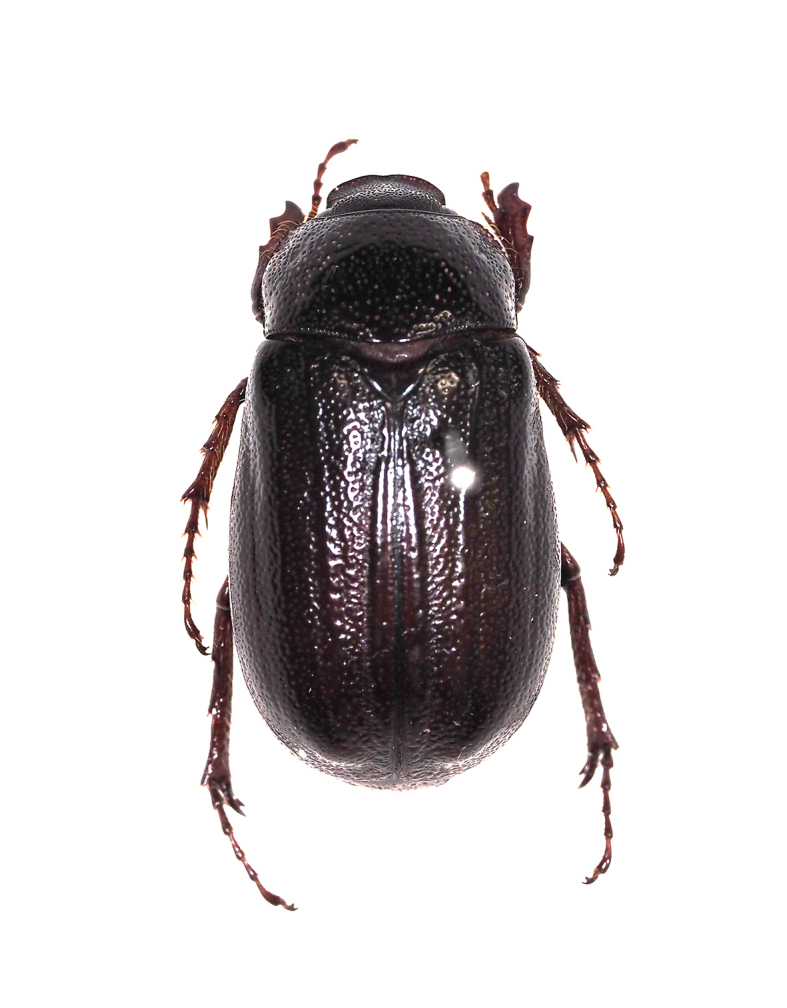
a black chafer (*Nigrotrichia kiotoensis*, Scarabaeidae).

**Figure 12.**
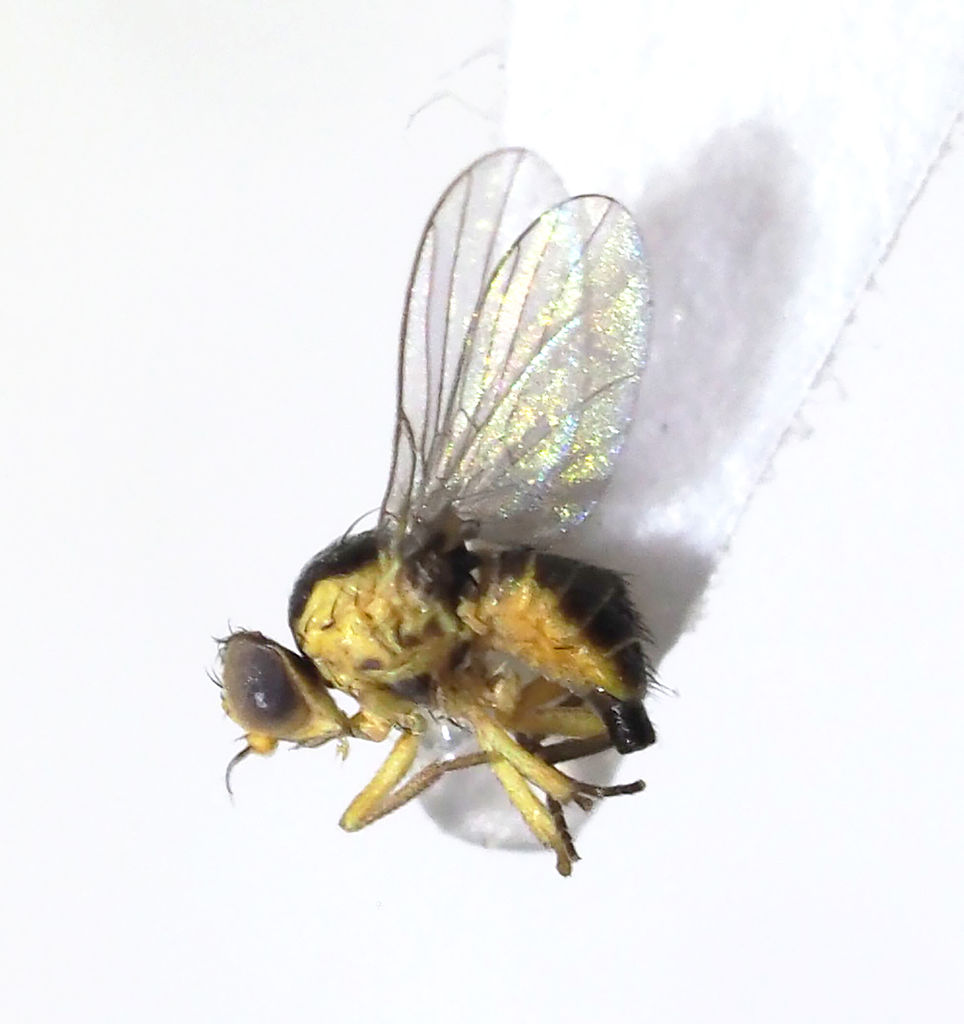
A vegetable leafminer (*Liriomyza sativae*, Agromyzidae) feeding on pepino.

**Figure 13a.**
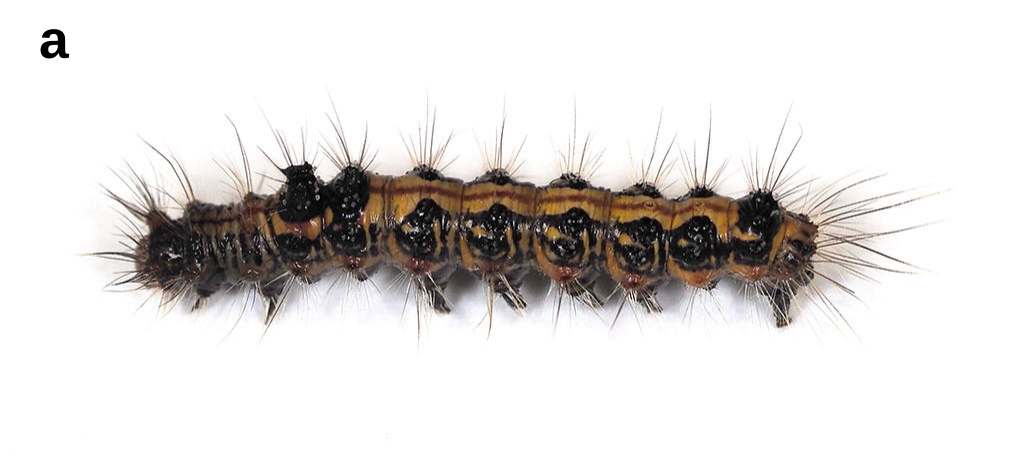
a tussock caterpillar (*Orvasca taiwana*, Lymantriidae).

**Figure 13b.**
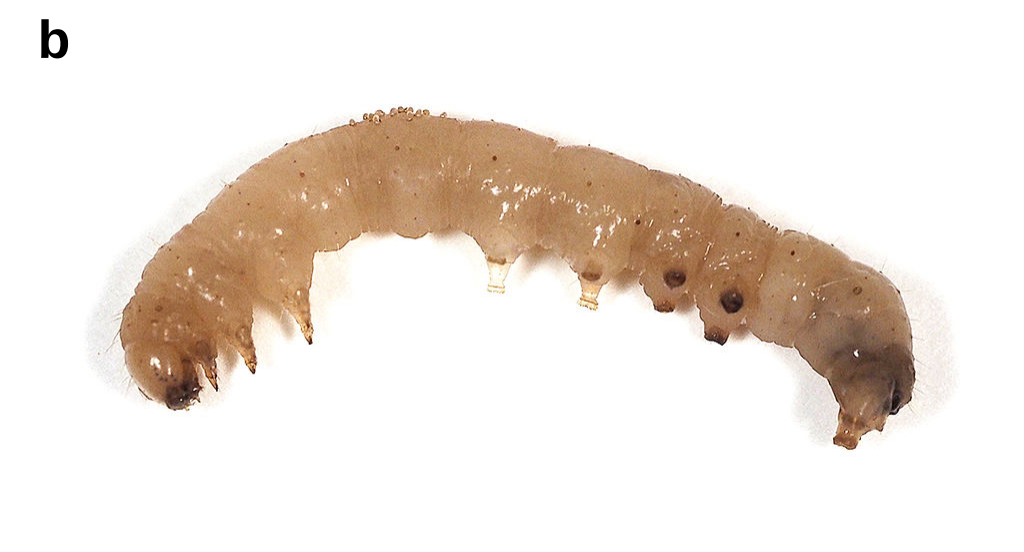
a hibiscus looper (*Gonitis mesogona*, Noctuidae).

**Figure 13c.**
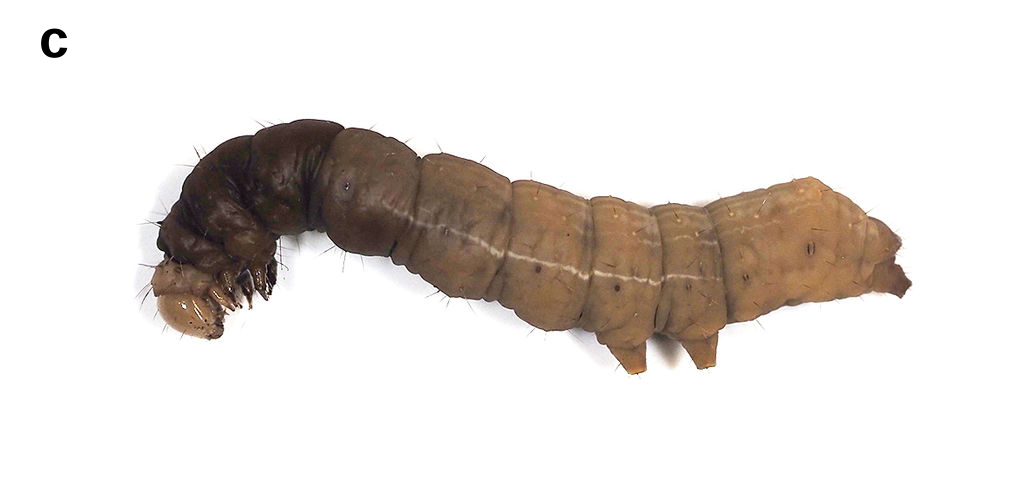
a cabbage looper (*Trichoplusia ni*, Noctuidae).

**Figure 13d.**
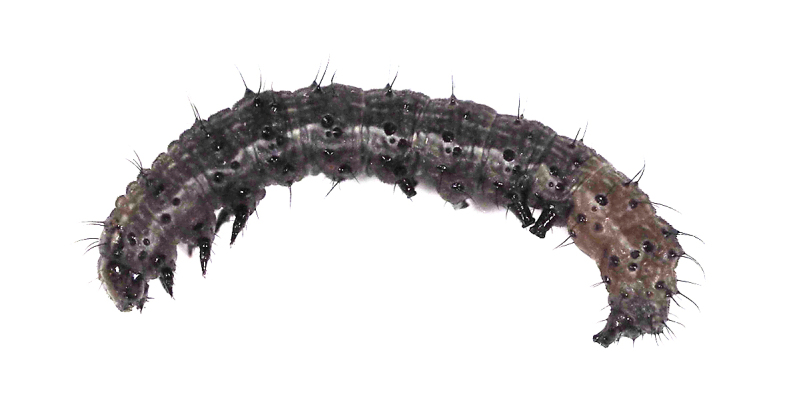
a tobacco budworm (*Helicoverpa armigera*, Noctuidae).

**Figure 13e.**
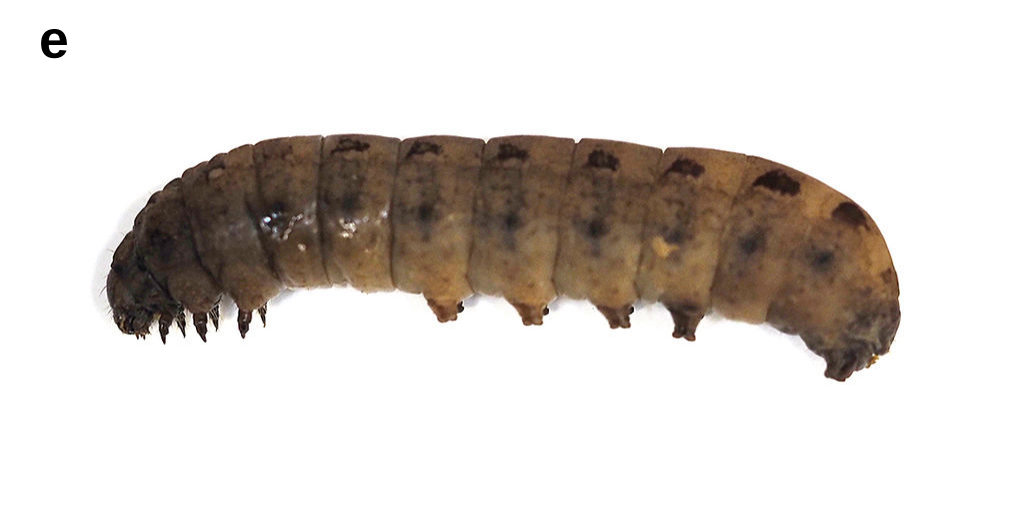
a tobacco cutworm (*Spodoptera litura*, Noctuidae).

**Figure 14a.**
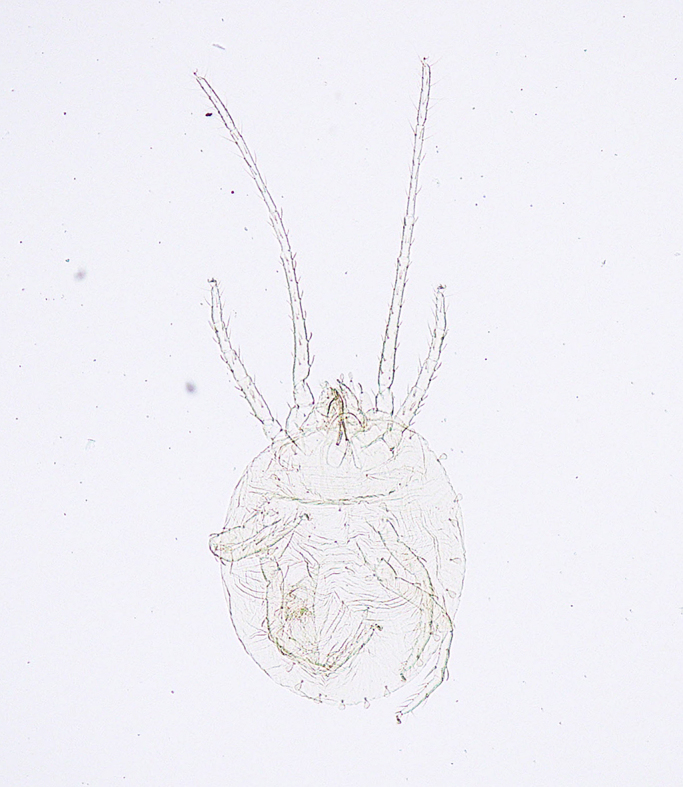
a clover mite (*Bryobia praetiosa*, Tetranychidae).

**Figure 14b.**
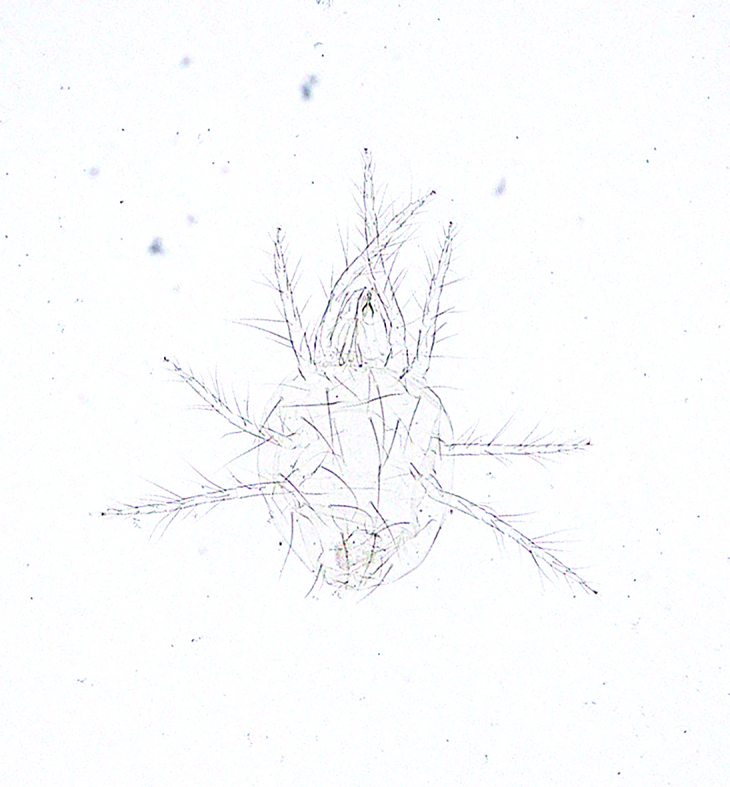
a tomato red spider mite (*Tetranychus evansi*, Tetranychidae).

**Figure 14c.**
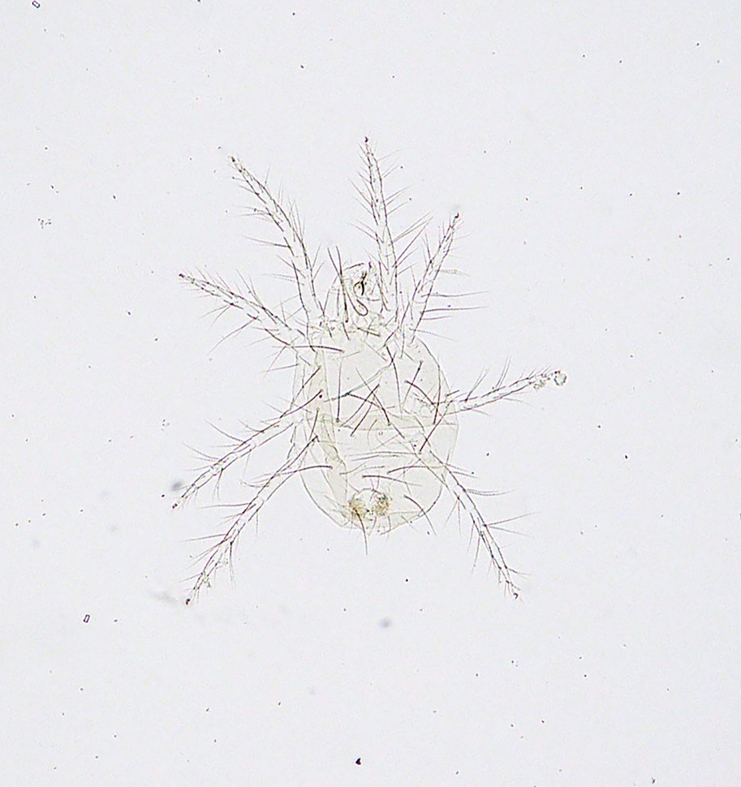
a *ludeni * spider mite (*Tetranychus ludeni*, Tetranychidae).

**Figure 14d.**
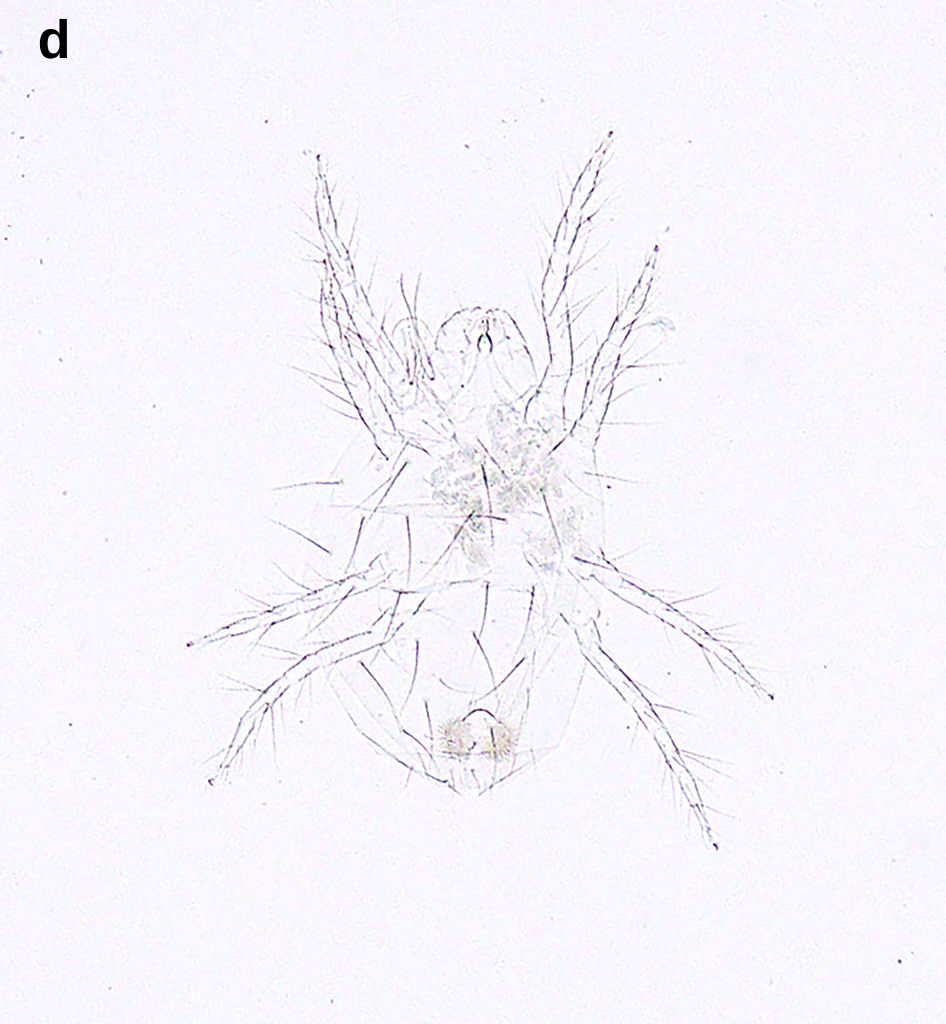
a two-spotted spider mite (*Tetranychus urticae*, Tetranychidae).

**Figure 15a.**
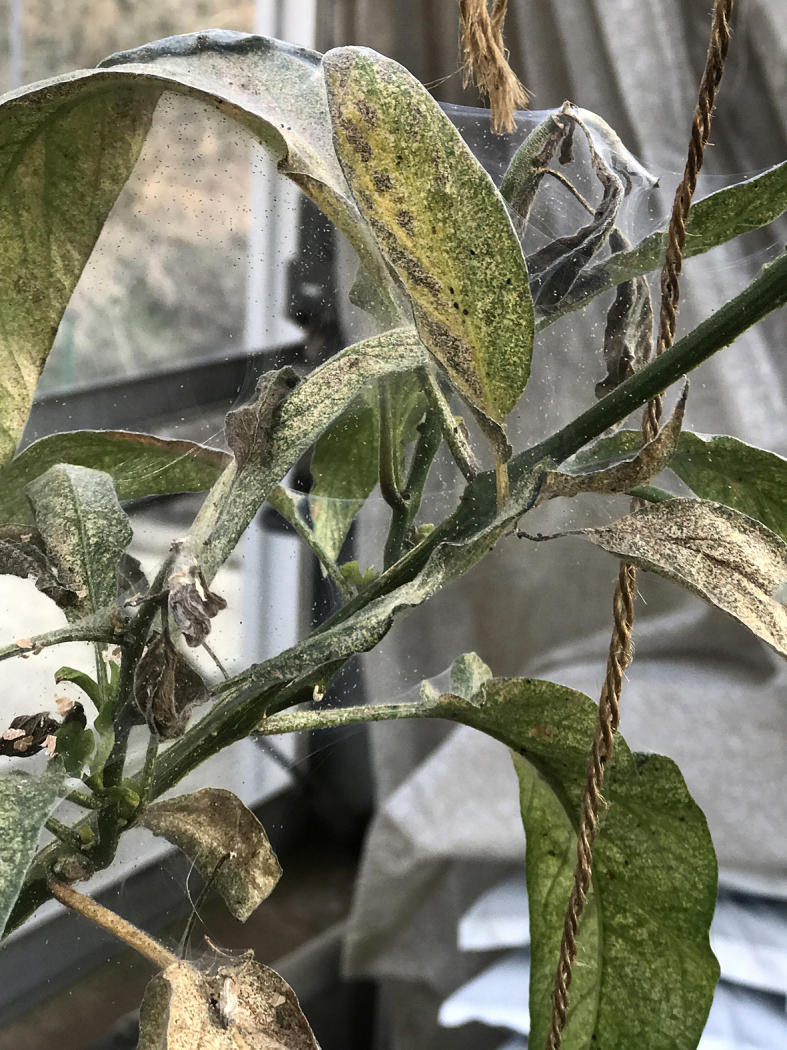
Pepino infested with two-spotted spider mites.

**Figure 15b.**
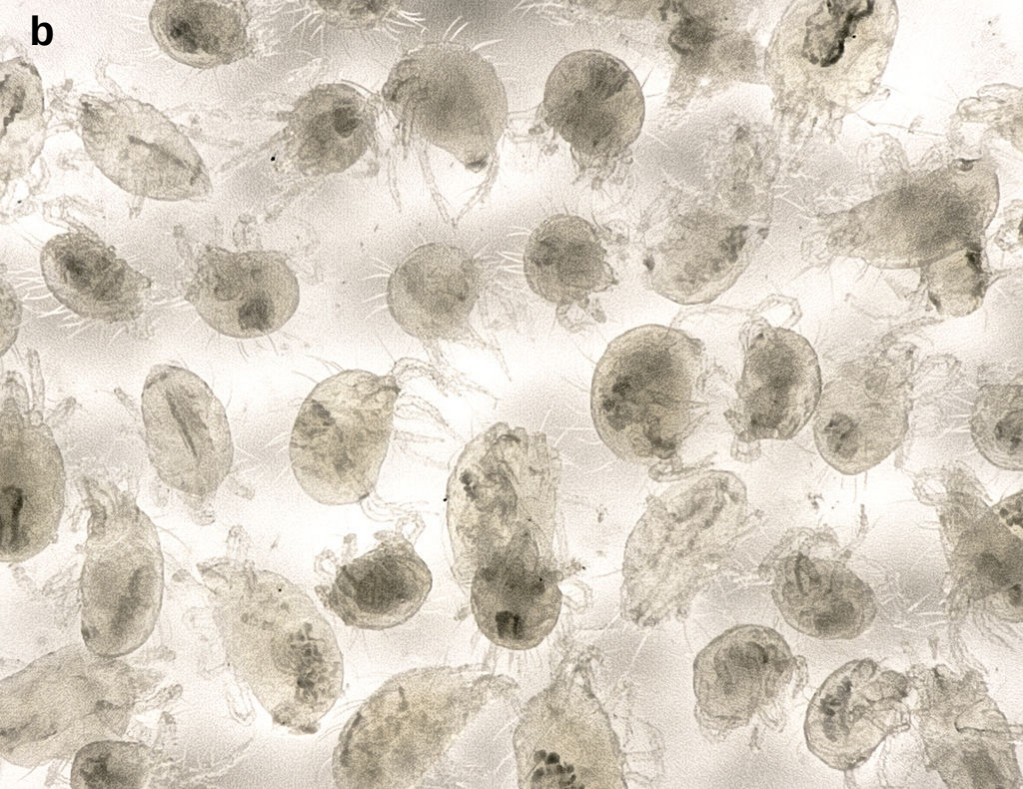
Two-spotted spider mites collected from pepino.

**Figure 16a.**
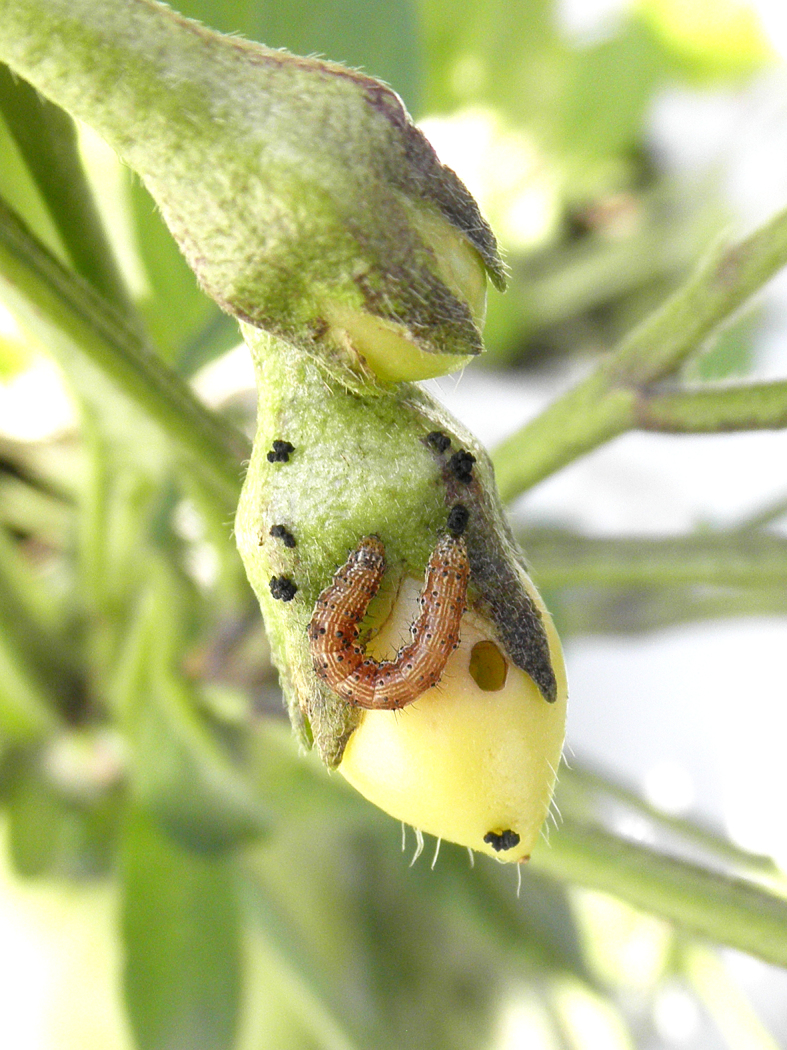
A tobacco budworm (*Helicoverpa armigera*, Noctuidae).

**Figure 16b.**
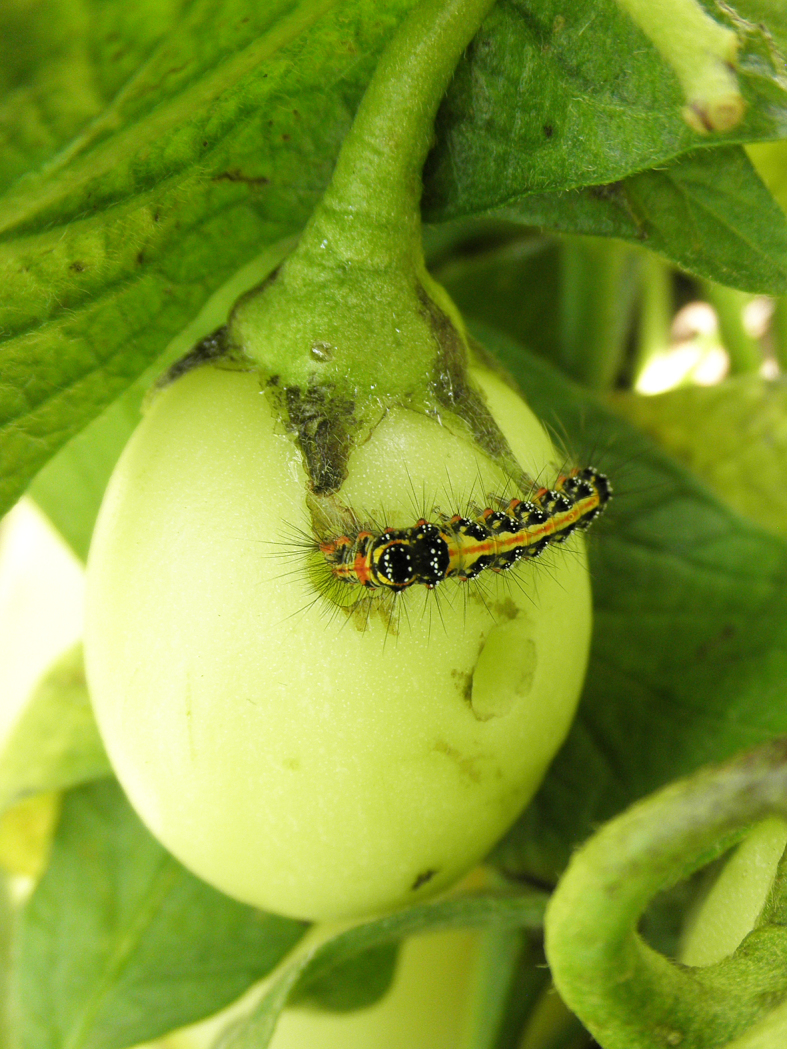
A tussock caterpillar (*Orvasca taiwana*, Lymantriidae).

**Table 1.** List of insect and mite pests found on pepino plants in Japan in the present study. The presence of the pests is indicated by "+".

Class, Order, Family	Species	Development stage	Feeding parts	Site 1	Site 2	Site 3	Site 4	Site 5	Site 6	Site 7	Site 8	Site 9	Site 10	Site 11
Entognatha, Collembola, Bourletiellidae	*Bourletiella hortensis* (Fitch, 1863) (Fig. 4)	adult	leaf		+									
Insecta, Orthoptera, Pyrgomorphidae	*Atractomorpha sinensis* Bolivar, 1905 (Fig. 5)	adult, nymph	leaf								+			
Insecta, Thysanoptera, Phlaeothripidae	*Haplothrips chinensis* Priesner, 1933 (Fig. 6a)	adult	leaf								+	+		
Insecta, Thysanoptera, Thripidae	*Frankliniella intonsa* (Trybom, 1895) (Fig. 6b)	adult	leaf			+								
Insecta, Thysanoptera, Thripidae	*Frankliniella occidentalis* (Pergande, 1895) (Fig. 6c)	adult	leaf	+		+				+	+			
Insecta, Thysanoptera, Thripidae	*Thrips nigropilosus* Uzel, 1895 (Fig. 6d)	adult	leaf		+	+								
Insecta, Thysanoptera, Thripidae	*Thrips palmi* Karny, 1925 (Fig. 6e)	adult	leaf								+	+	+	+
Insecta, Thysanoptera, Thripidae	*Thrips tabaci* Lindeman, 1889 (Fig. 6f)	adult	leaf	+	+	+		+			+	+	+	+
Insecta, Hemiptera, Aleyrodidae	*Bemisia tabaci* (Gennadius, 1889) (Fig. 7a)	adult, nymph	leaf		+	+		+			+		+	+
Insecta, Hemiptera, Aleyrodidae	*Trialeurodes vaporariorum* (Westwood, 1856) (Fig. 7b)	adult, nymph	leaf			+		+						
Insecta, Hemiptera, Aphididae	*Aphis gossypii* Glover, 1877 (Fig. 7c)	adult, nymph	leaf		+	+								
Insecta, Hemiptera, Aphididae	*Aphis spiraecola* Patch, 1914 (Fig. 7d)	adult	leaf									+		
Insecta, Hemiptera, Aphididae	*Macrosiphum euphorbiae* (Thomas, 1878) (Fig. 7e)	adult	leaf		+	+								
Insecta, Hemiptera, Aphididae	*Myzus persicae* (Sulzer, 1776) (Fig. 7f)	adult, nymph	leaf		+	+					+			
Insecta, Hemiptera, Cicadellidae	*Amrasca biguttula* (Ishida, 1913) (Fig. 8)	adult	leaf								+	+		
Insecta, Hemiptera, Tingidae	*Corythucha marmorata* (Uhler, 1878) (Fig. 9a)	adult	leaf		+		+							
Insecta, Hemiptera, Miridae	*Campylomma livida* Reuter, 1885 (Fig. 9b)	adult, nymph	leaf			+					+	+		
Insecta, Hemiptera, Miridae	*Prolygus bakeri* (Poppius, 1915) (Fig. 9c)	adult	leaf								+			
Insecta, Hemiptera, Miridae	*Taylorilygus apicalis* (Fieber, 1861) (Fig. 9d)	adult	leaf			+								
Insecta, Hemiptera, Pentatomidae	*Halyomorpha halys* (Stål, 1855) (Fig. 9e)	adult	leaf				+							
Insecta, Hemiptera, Coreidae	*Acanthocoris sordidus* (Thunberg, 1783) (Fig. 9f)	adult, nymph	stem		+									
Insecta, Coleoptera, Chrysomelidae	*Atrachya menetriesi* (Faldermann, 1835) (Fig. 10a)	adult	leaf		+									
Insecta, Coleoptera, Chrysomelidae	*Epitrix hirtipennis* (Melsheimer, 1847) (Fig. 10b)	adult	leaf		+	+								
Insecta, Coleoptera, Chrysomelidae	*Psylliodes angusticollis* Baly, 1874 (Fig. 10c)	adult	leaf									+		
Insecta, Coleoptera, Chrysomelidae	*Psylliodes punctifrons* Baly, 1874 (Fig. 10d)	adult	leaf		+									
Insecta, Coleoptera, Coccinellidae	*Henosepilachna vigintioctopunctata* (Fabricius, 1775) (Fig. 11a)	adult, larva	leaf									+		
Insecta, Coleoptera, Scarabaeidae	*Maladera orientalis* (Motschulsky, 1857) (Fig. 11b)	adult	leaf		+									
Insecta, Coleoptera, Scarabaeidae	*Nigrotrichia kiotoensis* (Niijima et Kinoshita, 1923) (Fig. 11c)	adult	leaf		+									
Insecta, Diptera, Agromyzidae	*Liriomyza sativae* Blanchard, 1938 (Fig. 12)	adult, larva	leaf					+						
Insecta, Lepidoptera, Lymantriidae	*Orvasca taiwana* (Shiraki, 1913) (Fig. 13a)	larva	leaf, fruit								+	+		
Insecta, Lepidoptera, Noctuidae	*Gonitis mesogona* (Walker, 1858) (Fig. 13b)	larva	leaf		+									
Insecta, Lepidoptera, Noctuidae	*Trichoplusia ni* (Hübner, 1803) (Fig. 13c)	larva	leaf			+					+	+		
Insecta, Lepidoptera, Noctuidae	*Helicoverpa armigera* (Hübner, 1808) (Fig. 13d)	larva	fruit								+			
Insecta, Lepidoptera, Noctuidae	*Spodoptera litura* (Fabricius, 1775) (Fig. 13e)	larva	leaf				+							
Arachnida, Trombidiformes, Tetranychidae	*Bryobia praetiosa* Koch, 1835 (Fig. 14a)	adult	leaf									+		
Arachnida, Trombidiformes, Tetranychidae	*Tetranychus evansi* Baker et Pritchard, 1960 (Fig. 14b)	adult	leaf								+			
Arachnida, Trombidiformes, Tetranychidae	*Tetranychus ludeni* Zacher, 1913 (Fig. 14c)	adult	leaf			+					+			
Arachnida, Trombidiformes, Tetranychidae	*Tetranychus urticae* Koch, 1836 (Fig. 14d)	adult, nymph	leaf	+	+	+		+	+				+	+

**Table 2.** Comprehensive list of insect and mite pests of pepino plants in Japan.

Class	Order	Family	Species	References
Entognatha	Collembola	Bourletiellidae	*Bourletiella hortensis* (Fitch, 1863)	present study
Insecta	Orthoptera	Pyrgomorphidae	*Atractomorpha sinensis* Bolivar, 1905	present study
Insecta	Thysanoptera	Phlaeothripidae	*Haplothrips chinensis* Priesner, 1933	present study
Insecta	Thysanoptera	Thripidae	*Frankliniella intonsa* (Trybom, 1895)	Kim et al. (2017), present study
Insecta	Thysanoptera	Thripidae	*Frankliniella occidentalis* (Pergande, 1895)	present study
Insecta	Thysanoptera	Thripidae	*Thrips nigropilosus* Uzel, 1895	present study
Insecta	Thysanoptera	Thripidae	*Thrips palmi* Karny, 1925	present study
Insecta	Thysanoptera	Thripidae	*Thrips tabaci* Lindeman, 1889	present study
Insecta	Hemiptera	Aleyrodidae	*Bemisia tabaci* (Gennadius, 1889)	Kim et al. (2017), present study
Insecta	Hemiptera	Aleyrodidae	*Trialeurodes vaporariorum* (Westwood, 1856)	Furusato (1984), Takahashi (1985), Takagi (1985), Kita (1986), Odagiri et al. (1986), Ozawa (1986), present study
Insecta	Hemiptera	Aphididae	*Aphis gossypii* Glover, 1877	Kim et al. (2017), present study
Insecta	Hemiptera	Aphididae	*Aphis spiraecola* Patch, 1914	present study
Insecta	Hemiptera	Aphididae	*Macrosiphum euphorbiae* (Thomas, 1878)	present study
Insecta	Hemiptera	Aphididae	*Myzus persicae* (Sulzer, 1776)	present study
Insecta	Hemiptera	Pseudococcidae	*Phenacoccus solani* Ferris, 1918	Kim et al. (2017)
Insecta	Hemiptera	Cicadellidae	*Amrasca biguttula* (Ishida, 1913)	present study
Insecta	Hemiptera	Tingidae	*Corythucha marmorata* (Uhler, 1878)	present study
Insecta	Hemiptera	Miridae	*Campylomma livida* Reuter, 1885	Kim et al. (2017), present study
Insecta	Hemiptera	Miridae	*Prolygus bakeri* (Poppius, 1915)	present study
Insecta	Hemiptera	Miridae	*Taylorilygus apicalis* (Fieber, 1861)	present study
Insecta	Hemiptera	Pentatomidae	*Halyomorpha halys* (Stål, 1855)	present study
Insecta	Hemiptera	Coreidae	*Acanthocoris sordidus* (Thunberg, 1783)	present study
Insecta	Coleoptera	Chrysomelidae	*Atrachya menetriesi* (Faldermann, 1835)	present study
Insecta	Coleoptera	Chrysomelidae	*Epitrix hirtipennis* (Melsheimer, 1847)	Kim et al. (2017), present study
Insecta	Coleoptera	Chrysomelidae	*Psylliodes angusticollis* Baly, 1874	present study
Insecta	Coleoptera	Chrysomelidae	*Psylliodes punctifrons* Baly, 1874	present study
Insecta	Coleoptera	Coccinellidae	*Henosepilachna vigintioctopunctata* (Fabricius, 1775)	present study
Insecta	Coleoptera	Scarabaeidae	*Maladera orientalis* (Motschulsky, 1857)	present study
Insecta	Coleoptera	Scarabaeidae	*Nigrotrichia kiotoensis* (Niijima et Kinoshita, 1923)	present study
Insecta	Diptera	Agromyzidae	*Liriomyza sativae* Blanchard, 1938	Kim et al. (2017), present study
Insecta	Lepidoptera	Gelechiidae	*Phthorimaea operculella* (Zeller, 1873)	Ozawa (1986)
Insecta	Lepidoptera	Lymantriidae	*Orvasca taiwana* (Shiraki, 1913)	present study
Insecta	Lepidoptera	Noctuidae	*Gonitis mesogona* (Walker, 1858)	present study
Insecta	Lepidoptera	Noctuidae	*Trichoplusia ni* (Hübner, 1803)	Kim et al. (2017), present study
Insecta	Lepidoptera	Noctuidae	*Helicoverpa armigera* (Hübner, 1808)	present study
Insecta	Lepidoptera	Noctuidae	*Spodoptera litura* (Fabricius, 1775)	Kim et al. (2017), present study
Arachnida	Acari	Tarsonemidae	*Polyphagotarsonemus latus* (Banks, 1904)	Kim et al. (2017)
Arachnida	Acari	Tetranychidae	*Bryobia praetiosa* Koch, 1835	present study
Arachnida	Acari	Tetranychidae	*Tetranychus evansi* Baker et Pritchard, 1960	present study
Arachnida	Acari	Tetranychidae	*Tetranychus ludeni* Zacher, 1913	present study
Arachnida	Acari	Tetranychidae	*Tetranychus urticae* Koch, 1836	Ozawa (1986), Kim et al. (2017), present study
